# Efficient penalized generalized linear mixed models for variable selection and genetic risk prediction in high-dimensional data

**DOI:** 10.1093/bioinformatics/btad063

**Published:** 2023-01-27

**Authors:** Julien St-Pierre, Karim Oualkacha, Sahir Rai Bhatnagar

**Affiliations:** Department of Epidemiology, Biostatistics and Occupational Health, McGill University, Montréal, QC H3A 1G1, Canada; Département de Mathématiques, Université du Québec à Montréal, Montréal, QC H2X 3Y7, Canada; Department of Epidemiology, Biostatistics and Occupational Health, McGill University, Montréal, QC H3A 1G1, Canada

## Abstract

**Motivation:**

Sparse regularized regression methods are now widely used in genome-wide association studies (GWAS) to address the multiple testing burden that limits discovery of potentially important predictors. Linear mixed models (LMMs) have become an attractive alternative to principal components (PCs) adjustment to account for population structure and relatedness in high-dimensional penalized models. However, their use in binary trait GWAS rely on the invalid assumption that the residual variance does not depend on the estimated regression coefficients. Moreover, LMMs use a single spectral decomposition of the covariance matrix of the responses, which is no longer possible in generalized linear mixed models (GLMMs).

**Results:**

We introduce a new method called pglmm, a penalized GLMM that allows to simultaneously select genetic markers and estimate their effects, accounting for between-individual correlations and binary nature of the trait. We develop a computationally efficient algorithm based on penalized quasi-likelihood estimation that allows to scale regularized mixed models on high-dimensional binary trait GWAS. We show through simulations that when the dimensionality of the relatedness matrix is high, penalized LMM and logistic regression with PC adjustment fail to select important predictors, and have inferior prediction accuracy compared to pglmm. Further, we demonstrate through the analysis of two polygenic binary traits in a subset of 6731 related individuals from the UK Biobank data with 320K SNPs that our method can achieve higher predictive performance, while also selecting fewer predictors than a sparse regularized logistic lasso with PC adjustment.

**Availability and implementation:**

Our Julia package PenalizedGLMM.jl is publicly available on github: https://github.com/julstpierre/PenalizedGLMM.

**Supplementary information:**

Supplementary data are available at *Bioinformatics* online.

## 1 Introduction

Genome-wide association studies (GWAS) have led to the identification of hundreds of common genetic variants, or single nucleotide polymorphisms (SNPs), associated with complex traits (Visscher *et al.*, 2017) and are typically conducted by testing association on each SNP independently. However, these studies are plagued with the multiple testing burden that limits discovery of potentially important predictors. Moreover, GWAS have brought to light the problem of missing heritability, that is, identified variants only explain a low fraction of the total observed variability for traits under study (Manolio *et al.*, 2009). Multivariable regression methods, on the other hand, simultaneously fit many SNPs in a single model and have been proposed to increase the power for identifying weaker associations compared to univariable methods (Li *et al.*, 2011). Moreover, sparse regularized multivariable regression models, which can perform variable selection, are exempt from the multiple testing burden.

Principal component analysis (PCA) can control for the confounding effect due to population structure by including the top eigenvectors of a genetic similarity matrix (GSM) as fixed effects in the regression model (Price *et al.*, 2006). Alternatively, using mixed models (MMs), one can model population structure and/or closer relatedness by including a polygenic random effect with variance–covariance structure proportional to the GSM (Yu *et al.*, 2006). Hence, while both PCA and MMs share the same underlying model, MMs are more robust in the sense that they do not require distinguishing between the different types of confounders (Price *et al.*, 2010). Moreover, MMs alleviate the need to evaluate the optimal number of principal components (PCs) to retain in the model as fixed effects.

Several authors have proposed to combine penalized quasi-likelihood (PQL) estimation with sparsity inducing regularization to perform selection of fixed and/or random effects in generalized linear mixed model (GLMM) (Groll and Tutz, 2014; Hui *et al.*, 2017). However, none of these methods are currently scalable for modern large-scale genome-wide data, nor can they directly incorporate relatedness structure through the use of a kinship matrix. Indeed, the computational efficiency of recent multivariable methods for high-dimensional MMs rely on performing a spectral decomposition of the covariance matrix to rotate the phenotype and design matrix such that the transformed data become uncorrelated (Bhatnagar *et al.*, 2020b; Rakitsch *et al.*, 2013). These methods are typically restricted to linear models since in GLMMs, it is no longer possible to perform a single spectral decomposition to rotate the phenotype and design matrix, as the covariance matrix depends on the sample weights which in turn depend on the estimated regression coefficients that are being iteratively updated. This limits the application of high-dimensional MMs to analysis of binary traits in genetic association studies.

In this article, we introduce a new method called pglmm that allows to simultaneously select variables and estimate their effects, accounting for between-individual correlations and binary nature of the trait. We develop a scalable algorithm based on PQL estimation which makes it possible to fit penalized GLMMs on high-dimensional GWAS of binary traits. To speedup computation, we estimate the variance components and dispersion parameter of the model under the null hypothesis of no genetic effect. Secondly, we use an upper-bound for the inverse variance–covariance matrix in order to perform a single spectral decomposition of the GSM and greatly reduce memory usage. Finally, we implement an efficient cyclic coordinate descent algorithm in order to find the optimal estimates for the fixed and random effects parameters. Our method is implemented in an open source Julia programming language (Bezanson *et al.*, 2017) package called PenalizedGLMM.jl and freely available at https://github.com/julstpierre/PenalizedGLMM.

The rest of this article is structured as follows. In Section 2, we present our model, describe the cyclic coordinate descent algorithm that is used to estimate the parameters and detail how predictions are obtained in GLMs with PC adjustment versus our proposed mixed model. In Section 3, we show through simulations that both LMM and logistic model with PC adjustment fail to correctly select important predictors and estimate their effects when the dimensionality of the kinship matrix is high. Further, we demonstrate through the analysis of two polygenic binary traits in a subset of 6731 related individuals from the UK Biobank data that our method achieves higher predictive performance, while also selecting consistently fewer predictors than a logistic lasso with PC adjustment. We finish with a discussion of our results, some limitations and future directions in Section 4.

## 2 Materials and methods

### 2.1 Model

We consider the following GLMM

(1) $$g(\mu_{i})=\eta_{i}=X_{i}\alpha +G_{i}\gamma +b_{i},$$

for i=1,..,n, where $\mu_{i}=E(y_{i}|X_{i},G_{i},b_{i}),\;X_{i}$ is a 1×m row vector of covariates for subject *i*, α is a m×1 column vector of fixed covariate effects including the intercept, $G_{i}$ is a 1×p row vector of genotypes for subject *i* taking values {0, 1, 2} as the number of copies of the minor allele, and γ is a p×1 column vector of fixed additive genotype effects. We assume that $b={\left(b_{1},\ldots ,b_{n}\right)}^{⊺}∼N(0,\sum_{s=1}^{S}\tau_{s}V_{s})$ is an n×1 column vector of random effects, $\tau ={\left(\tau_{1},\ldots ,\tau_{S}\right)}^{⊺}$ are variance component parameters, $V_{1}$ is a known kinship matrix or GSM typically estimated from high-quality common genotype markers (MAF ≥0.01) (Yang *et al.*, 2011) and $V_{2},\ldots ,V_{S}$ are any known *n *×* n* positive semi-definite matrices to account for shared environmental effects or complex sampling designs. The phenotypes *y_i_* are assumed to be conditionally independent and identically distributed given $\left(X_{i},G_{i},b\right)$ and follow any exponential family distribution with canonical link function g(·), mean $E(y_{i}|b)=\mu_{i}$ and variance $\text{Var}(y_{i}|b)=\phi a_{i}^{-1}\nu (\mu_{i}),$ where ϕ is a dispersion parameter, *a_i_* are known weights and ν(·) is the variance function. In order to estimate the parameters of interest and perform variable selection, we need to use an approximation method to obtain a closed analytical form for the marginal likelihood of model (1). Following the derivation of Chen *et al.* (2016), we propose to fit (1) using a PQL method, from where the log integrated quasi-likelihood function is equal to

(2) $$\begin{array}{c} ql(\alpha ,\gamma ,\phi ,\tau )=-\frac{1}{2}\text{log}\;\left|\sum_{s=1}^{S}\tau_{s}V_{s}W+I_{n}\right|+\sum_{i=1}^{n}ql_{i}(\alpha ,\gamma |\tilde{b})\\ -\frac{1}{2}{\tilde{b}}^{⊺}{\left(\sum_{s=1}^{S}\tau_{s}V_{s}\right)}^{-1}\tilde{b}, \end{array}$$

where $W=\text{diag}\left\{\frac{a_{i}}{\phi \nu (\mu_{i})[g\prime {\left(\mu_{i}\right)}^{2}]}\right\}$ is a diagonal matrix containing weights for each observation, $ql_{i}(\alpha ,\gamma |b)=\int_{y_{i}}^{\mu_{i}}\frac{a_{i}(y_{i}-\mu )}{\phi \nu (\mu )}d\mu$ is the quasi-likelihood for the *i*th individual given the random effects b, and $\tilde{b}$ is the solution which maximizes (2).

In typical genome-wide studies, the number of predictors is much greater than the number of observations (*p *>* n*), and the parameter vector γ becomes underdetermined when modeling SNPs jointly. Thus, we propose to add a lasso regularization term (Tibshirani, 1996) to the negative quasi-likelihood function in (2) to seek a sparse subset of γ that gives an adequate fit to the data. Because ql(α,γ,ϕ,τ) is a non-convex loss function, we propose a two-step estimation method to reduce the computational complexity. First, we obtain the variance component estimates $\hat{\phi }$ and $\hat{\tau }$ under the null hypothesis of no genetic effect (γ=0) using the AI-REML algorithm (Gilmour *et al.*, 1995) detailed in Supplementary Appendix SA. Second, assuming that the weights in ***W*** vary slowly with the conditional mean, we drop the first term in (2) (Breslow and Clayton, 1993) and define the following objective function which we seek to minimize with respect to $\left(\alpha ,\gamma ,\tilde{b}\right)$:

(3) $$\begin{array}{c} (\hat{\alpha },\hat{\gamma },\hat{b})=\underset{\alpha ,\gamma ,\tilde{b}}{\text{argmin}\;}Q_{\lambda }(\alpha ,\gamma ,\tilde{b}),\\ Q_{\lambda }(\alpha ,\gamma ,\tilde{b})=-\sum_{i=1}^{n}ql_{i}(\alpha ,\gamma |\tilde{b})+\frac{1}{2}{\tilde{b}}^{⊺}{\left(\sum_{s=1}^{S}{\hat{\tau }}_{s}V_{s}\right)}^{-1}\tilde{b}+\lambda \sum_{j}v_{j}|\gamma_{j}|\\ :=-\ell_{\mathrm{PQL}}(\alpha ,\gamma ,\hat{\phi },\hat{\tau }|\tilde{b})+\lambda \sum_{j}v_{j}|\gamma_{j}|, \end{array}$$

where *λ* is a non-negative regularization parameter, and *v_j_* is a penalty factor for the *j*th predictor. This two-step approach is known as the P3D (population parameters previously determined) method (Zhang *et al.*, 2010), which is a common approach in mixed-model association tests, and it has been shown to outperform both PCA and genomic control in correcting for sample structure (Kang *et al.*, 2010). Moreover, Reisetter and Breheny (2021) showed through simulation studies that in the case of penalized LMMs, estimating the variance components once performed similarly in terms of estimating the SNP coefficients than by including the variance components in the iterative procedure, while showing much greater computational efficiency and numerical stability. By default, we standardize the genotype counts and assign *v_j_* = 1 for all genetic predictors in (3), which is equivalent to using unscaled genotypes with $v_{j}^{-1}=\sqrt{2{\text{MAF}}_{j}(1-{\text{MAF}}_{j})}$ where the MAFs are estimated from the data. Alternatively, it is possible to use an adaptive lasso penalty with weights $v_{j}=|{\hat{\beta }}_{j}{|}^{-\kappa }$, where *κ* is a common power parameter and ${\hat{\beta }}_{j}$ is the coefficient estimate obtained by univariable marginal regression (Waldmann *et al.*, 2019).

In Supplementary Appendix SB, we detail our proposed cyclic coordinate gradient descent algorithm to solve (3) and obtain regularized PQL estimates for $\beta ={\left(\alpha^{⊺},\gamma^{⊺}\right)}^{⊺}$ and $\tilde{b}$. Briefly, our algorithm is equivalent to iteratively solving the two penalized weighted least squares (WLS)

$$\underset{\tilde{b}}{\text{argmin}}{\left(\tilde{Y}-\tilde{X}\beta -\tilde{b}\right)}^{⊺}W\left(\tilde{Y}-\tilde{X}\beta -\tilde{b}\right)+{\tilde{b}}^{⊺}{\left(\sum_{s=1}^{S}{\hat{\tau }}_{s}V_{s}\right)}^{-1}\tilde{b},$$

and

(4) $$\underset{\beta }{\text{argmin}}{\left(\tilde{Y}-\tilde{X}\beta \right)}^{⊺}\Sigma^{-1}\left(\tilde{Y}-\tilde{X}\beta \right)+\lambda \sum_{j}v_{j}|\beta_{j}|,$$

where $\Sigma =W^{-1}+\sum_{s=1}^{S}{\hat{\tau }}_{s}V_{s}$ is the covariance matrix of the working response vector $\tilde{Y}$, and $\tilde{X}=\left[X;\;G\right]$. We use the spectral decomposition of Σ to rotate $\tilde{Y},\;\tilde{X}$ and $\tilde{b}$ in (4) such that the transformed data is uncorrelated. Given the current estimate for $\beta ,\;\tilde{b}$ can be shown to be equal to a generalized ridge-like WLS estimator with $\tilde{X}\beta$ as an offset. Hence, by profiling out $\tilde{b}$ from the objective function and replacing it by its closed-form estimate, we estimate β by cycling through its coordinates and minimizing the objective function with respect to one coordinate at a time. In this work, we focus on penalized GLMMs for high-dimensional (*p *>* n*) GWAS data of binary traits, for which we can use a lower bound on Σ so that a single spectral decomposition is performed (Böhning and Lindsay, 1988). Although the methods apply to GLMMs for any exponential family, e.g. counts following a Poisson distribution, we need to perform a spectral decomposition of Σ each time we update the weight matrix ***W***. Hence, for other distributions, further work is needed to address these computational limitations for application to high-dimensional GWAS data. All calculations and algorithmic steps are detailed in Supplementary Appendix SB.

### 2.2 Prediction

It is often of interest in genetic association studies to make predictions on a new set of individuals, e.g. the genetic risk of developing a disease for a binary response or the expected outcome in the case of a continuous response. In what follows, we compare how predictions are obtained using pglmm versus a GLM with PC adjustment.

#### 2.2.1 pglmm

Suppose a single variance component is needed such that $b∼N(0,\tau_{1}V_{1})$ where $V_{1}$ is the GSM between *n* subjects that are used to fit the GLMM (1). We iteratively fit on a training set of size *n* the working linear mixed model

$$\tilde{Y}=\tilde{X}\beta +b+\epsilon ,$$

where $\epsilon =g\prime (\mu )(y-\mu )∼N(0,W^{-1})$. Let ${\tilde{Y}}_{s}$ be the latent working vector in a testing set of *n_s_* individuals with predictor set ${\tilde{X}}_{s}$. Similar to Bhatnagar *et al.* (2020b), we assume that the marginal joint distribution of ${\tilde{Y}}_{s}$ and $\tilde{Y}$ is multivariate Normal:

$$\left[\begin{array}{c} {\tilde{Y}}_{s}\\ \tilde{Y} \end{array}\right]∼N\left(\left[\begin{array}{c} {\tilde{X}}_{s}\beta \\ \tilde{X}\beta \end{array}\right],\left[\begin{array}{cc} \Sigma_{11} & \Sigma_{12}\\ \Sigma_{21} & \Sigma_{22} \end{array}\right]\right),$$

where $\Sigma_{12}=\tau_{1}V_{12}$ and $V_{12}$ is the $n_{s}\times n$ GSM between the testing and training individuals. It follows from standard normal theory that

$$\begin{array}{c} {\tilde{Y}}_{s}|\tilde{Y},\phi ,\tau_{1},\beta ,\tilde{X},{\tilde{X}}_{s}∼\\ \;\;N\left({\tilde{X}}_{s}\beta +\Sigma_{12}\Sigma_{22}^{-1}(\tilde{Y}-\tilde{X}\beta ),\Sigma_{11}-\Sigma_{12}\Sigma_{22}^{-1}\Sigma_{21}\right). \end{array}$$

The predictions are based on the conditional expectation $E[{\tilde{Y}}_{s}|\tilde{Y},\phi ,\tau_{1},\beta ,\tilde{X},{\tilde{X}}_{s}]$, that is

(5) $$\begin{array}{c} {\hat{\mu }}_{s}=g^{-1}\left(E[{\tilde{Y}}_{s}|\tilde{Y},\hat{\phi },{\hat{\tau }}_{1},\hat{\beta },\tilde{X},{\tilde{X}}_{s}]\right)\\ =g^{-1}\left({\tilde{X}}_{s}\hat{\beta }+{\hat{\tau }}_{1}V_{12}{\left(W^{-1}+{\hat{\tau }}_{1}V_{1}\right)}^{-1}(\tilde{Y}-\tilde{X}\hat{\beta })\right)\\ =g^{-1}\left({\tilde{X}}_{s}\hat{\beta }+V_{12}U{\left(\frac{1}{{\hat{\tau }}_{1}}D^{-1}+U^{⊺}\mathrm{WU}\right)}^{-1}U^{⊺}W(\tilde{Y}-\tilde{X}\hat{\beta })\right), \end{array}$$

where g(·) is the link function and U is the *n *×* n* matrix of PCs obtained from the spectral decomposition of the GSM for training subjects.

#### 2.2.2 GLM with PC adjustment

Another approach to control for population structure and/or subjects’ relatedness is to use the first *r* columns of U as unpenalized fixed effects covariates (Privé *et al.*, 2020). This leads to the following GLM

$$g(\mu )=\tilde{X}\beta +U_{r}\delta ,$$

where $U_{r}$ is the *n *×* r* design matrix for the first *r* PCs and $\delta \in R^{r}$ is the corresponding vector of fixed effects. Letting $\tilde{Y}=\tilde{X}\beta +U_{r}\delta +g\prime (\mu )(y-\mu )$ be the working response vector, one can show that

(6) $$\hat{\delta }={\left(U_{r}^{⊺}WU_{r}\right)}^{-1}U_{r}^{⊺}W\left(\tilde{Y}-\tilde{X}\hat{\beta }\right),$$

where ***W*** is the diagonal matrix of GLM weights. Recall that $V_{12}$ is the $n_{s}\times n$ GSM between the test and training sets subjects such that the projected PCs on the testing subjects are equal to $V_{12}U_{r}$. Then, the estimated mean response ${\hat{\mu }}_{s}$ for the testing set is given by

(7) $$\begin{array}{c} {\hat{\mu }}_{s}=g^{-1}\left(\tilde{X_{s}}\hat{\beta }+V_{12}U_{r}\hat{\delta }\right)\\ =g^{-1}\left(\tilde{X_{s}}\hat{\beta }+V_{12}U_{r}{\left(U_{r}^{⊺}WU_{r}\right)}^{-1}U_{r}^{⊺}W\left(\tilde{Y}-\tilde{X}\hat{\beta }\right)\right). \end{array}$$

By comparing (5) and (7), we see that both GLM with PC adjustment and pglmm use a projection of the training PCs on the testing set to predict new responses, but with different coefficients for the projected PCs. For the former, the estimated coefficients for the first *r* projected PCs in (6) are obtained by iteratively solving generalized least squares (GLS) on the partial working residuals $\tilde{Y}-\tilde{X}\hat{\beta }$. For pglmm, the estimated coefficients for all projected PCs are also obtained by iteratively solving GLS on the partial working residuals $\tilde{Y}-\tilde{X}\hat{\beta }$, with an extra ridge penalty for each coefficient that is equal to ${\hat{\tau_{1}}}^{-1}\Lambda_{i}^{-1}$ with Λ_*i*_ the *i*th eigenvalue of ***V*** that is associated with the *i*th PC.

Hence, pglmm shrinks PCs coefficients proportionally to their corresponding eigenvalues in a smooth way, while the fixed effect GLM uses a thresholding approach; the first *r* predictors with larger eigenvalues are kept intact, and the others are completely removed. This implies that the confounding effect from population structure and/or relatedness on the phenotype is fully captured by the first *r* PCs. As we show in simulations, departure from this assumption may lead to higher false-positive rates and decrease prediction accuracy.

### 2.3 Simulation design

We evaluated the performance of our proposed method against that of a lasso LMM, using the R package ggmix (Bhatnagar *et al.*, 2020a), and a logistic lasso, using the Julia package GLMNet which wraps the Fortran code from the original R package glmnet (Friedman *et al.*, 2010). We compared glmnet when we included or not the first 10 PCs in the model (glmnetPC). We performed a total of 50 replications for two simulation scenarios, drawing anew genotypes and simulated traits. Values for all simulation parameters are presented in Table 1.

**Table 1.** Values for all simulation parameters

Parameter	Definition	Scenario 1	Scenario 2
		BN-PSD model	Real genotype
*M*	Number of replications	50	50
$h_{g}^{2}$	Fraction of variance due to fixed genetic effects	0.5	0.17
$h_{b}^{2}$	Fraction of variance due to random genetic effects	0.4	0.4
*π*_0_	Prevalence under the null	0.1	0.1
*n*	Sample size	2500	6731
*p*	Number of SNPs	5000	15 000
*c*	Fraction of causal SNPs	1%	1%

*Note*: In the first scenario, we simulated binary phenotypes and random genotypes from the BN-PSD admixture model using the bnpsd package in R. In the second scenario, we simulated binary phenotypes using a total of 6731 subjects of White British ancestry from the UK Biobank data having estimated first, second or third degree relationships with at least one other individual.

#### 2.3.1 Simulated genotype from the admixture model

In the first scenario, we studied the performance of all methods for different population structures by simulating random genotypes from the BN-PSD admixture model for 10 or 20 subpopulations with 1D geography or independent subpopulations using the bnpsd package in R (Ochoa and Storey, 2021). Sample size was set to *n *=* *2500. We simulated *p *=* *5000 candidate SNPs and randomly selected *c* = 1% to be causal. The kinship matrix ***V*** and PCs were calculated using a set of 50 000 additional simulated SNPs. We simulated covariates for age and sex using Normal and Binomial distributions, respectively.

For each replication, subjects were partitioned into training and test sets using an 80/20 ratio. Variable selection and coefficient estimation were performed on training subjects for all methods. We compared each method at a fixed number of active predictors, ranging from 5 to either 50 which corresponds to the number of true causal SNPs. Comparisons were based on three criteria: the ability to retrieve the causal predictors, measured by the true positive rate

$$\text{TPR}=\frac{\left|\{1\leq k\leq p:{\hat{\beta }}_{k}\neq 0\cap \beta_{k}\neq 0\}\right|}{\left|\{1\leq k\leq p:\beta_{k}\neq 0\}\right|},$$

the ability to accurately estimate coefficients, measured by the root mean squared error

$$\text{RMSE}=\sqrt{\frac{1}{p}\sum_{k=1}^{p}{\left({\hat{\beta }}_{k}-\beta_{k}\right)}^{2}},$$

and the ability to predict outcomes in the test sets, measured by the area under the roc curve (AUC).

#### 2.3.2 Real genotypes from the UK Biobank data

In the second scenario, we compared the performance of all methods when a high proportion of related individuals are present, using real genotype data from the UK Biobank. We retained a total of 6731 subjects of White British ancestry having estimated first, second or third degree relationships with at least one other individual. We compared methods in a more realistic setting with weaker effect sizes and more causal variants than the first scenario. We sampled p=15 000 candidate SNPs among all chromosomes and randomly selected *c* = 1% to be causal. We used PCs as provided with the dataset. These were computed using a set of unrelated samples and high-quality markers pruned to minimize LD (Bycroft *et al.*, 2018). Then, all subjects were projected onto the principal components using the corresponding loadings. Since the markers that were used to compute the PCs were potentially sampled as candidate causal markers in our simulations, we included all candidate SNPs in the set of markers used for calculating the kinship matrix ***V***. We simulated age using a Normal distribution and used the sex covariate provided with the data.

For this simulation scenario, we evaluated the performance of all methods when using cross-validation as a model selection criteria, rather than fixing the number of active predictors in the model. For this, the 6731 subjects from the UK Biobank data were randomly split into training (40%), validation (30%) and test (30%) sets, ensuring all related individuals were assigned into the same set. For cross-validation, the full lasso solution path was fitted on the training set, and the regularization parameter was obtained on the model which maximized AUC on the validation set. We also evaluated the performance of our proposed method when using AIC as a model selection criterion. Again, we compared methods performance on the basis of TPR, AUC on the test sets and RMSE. In addition, we compared each model selection approach on the total number of predictors selected and on the model precision, which is defined as the proportion of selected predictors that are true positives.

#### 2.3.3 Simulation model

Let *S* be the set of candidate causal SNPs, with |S|=p×c, then the causal SNPs fixed effects *β_j_* were generated from a Gaussian distribution $N(0,h_{g}^{2}\sigma^{2}/|S|)$, where $h_{g}^{2}$ is the fraction of variance on the logit scale that is due to total additive genetic fixed effects. That is, we assumed the candidate causal markers explained a fraction of the total polygenic heritability, and the rest was explained by a random polygenic effect $b∼N(0,h_{b}^{2}\sigma^{2}V)$. For the first scenario, we simulated a signal-to-noise ratio (SNR) equal to 1 for the fixed genetic effects ($h_{g}^{2}=50\%$) under strong random polygenic effects ($h_{b}^{2}=40\%$). For the second scenario, we simulated fixed effects using $h_{g}^{2}=17\%$, which corresponds to the estimated SNP heritability for asthma on the liability scale (see https://nealelab.github.io/UKBB_ldsc/h2_summary_20002_1111.html), again under strong random polygenic effects ($h_{b}^{2}=40\%$). We then simulated a binary phenotype using a logistic link function

(8) $$\begin{array}{c} \text{logit}(\pi )=\text{logit}(\pi_{0})-\text{log}(1.3)\times \text{Sex}+\text{log}(1.05)\text{Age}/10\\ \;\;+\sum_{j\in S}\beta_{j}\cdot {\tilde{G}}_{j}+b, \end{array}$$

where the parameter *π*_0_ was chosen to specify the prevalence under the null, and ${\tilde{G}}_{j}$ is the *j*th column of the standardized genotype matrix ${\tilde{g}}_{ij}=(g_{ij}-2p_{i})/\sqrt{2p_{i}(1-p_{i})}$ and *p_i_* is the MAF. By using the spectral decomposition of the kinship matrix ***V***, we can show that b=Uδ, where $\delta ∼N(0,h_{b}^{2}\sigma^{2}D)$, ***U*** is the *n *×* n* matrix of PCs, and ***D*** is a diagonal matrix of corresponding eigenvalues. Thus, the unmeasured confounding effect δ is correlated with the population structure through the design matrix of PCs ***U***.

### 2.4 Real data application

We used the same set of 6731 related subjects from the UK Biobank dataset presented in Section 2.3.2 to construct a polygenic risk score (PRS) on two highly heritable binary traits, asthma (self-reported, UK Biobank code: 20002_1111) and high cholesterol (self-reported, UK Biobank code: 20002_1473). We present demographics and number of cases for both analyses in Table 2. After filtering for SNPs with missing rate smaller than 0.01, MAF above 0.05 and a *P*-value for the Hardy–Weinberg exact test above $10^{-6}$, a total of 320K genotype SNPs were remaining.

**Table 2.** Demographics for the real data application

	Asthma		High cholesterol	
	Cases	Controls	Cases	Controls
*N* (%)	819 (12.2)	5912 (87.8)	883 (13.1)	5848 (86.7)
Age Median (IQR)	58 (16)	59 (15)	64 (7)	57 (16)
Male (%)	306 (37.4)	2571 (43.5)	467 (52.9)	2410 (41.2)

*Note*: We retained a total of 6731 subjects of White British ancestry from the UK Biobank data having estimated first, second or third degree relationships with at least one other individual.

To better understand the contribution of the PRS for predicting asthma and high cholesterol, we fitted for each trait a null model with only age, sex, genotyping platform and the first 10 PCs as fixed effects. Since for highly polygenic traits, it is generally considered that there are a large number of predictors with small to moderate effects (O’Connor *et al.*, 2019), we also fitted a standard genomic best linear unbiased prediction (gBLUP) model (Ødegård *et al.*, 2018). The gBLUP model corresponds to the null model of pglmm, i.e. a model where we include age, sex and genotyping platform as fixed effects, and one random effect with variance–covariance proportional to the GSM. For both gBLUP and pglmm, we did not include any PC since kinship is accounted for by the random effect. Finally, we also fitted a logistic lasso in which the top 10 PCs were included as unpenalized covariates in addition to age, sex and genotyping platform (glmnetPC). To evaluate the predictive performance of the compared methods in independent subjects, we randomly split the subjects in training (80%) and test (20%) sets for a total of 40 times. For each of the 40 replications, the full lasso solution path was fitted on the training set only. For pglmm, the regularization parameter (*λ*) was selected to minimize the AIC on the training data. For glmnetPC, the regularization parameter was obtained by minimizing the deviance using 10-fold cross-validation on the training data. We compared mean prediction accuracy on the test sets as well as the median number of predictors included in all models.

## 3 Results

### 3.1 Simulation results for the first scenario

**Fig. 1.**
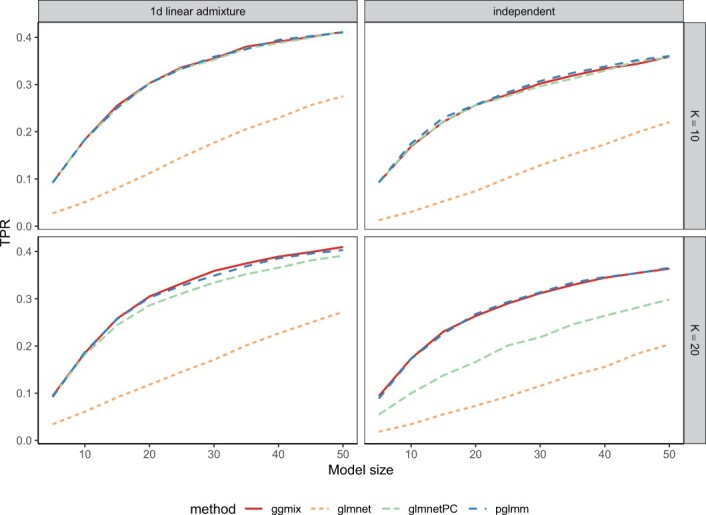
Mean of 50 TPRs for the first simulation scenario where we simulated random genotypes from the BN-PSD admixture model. *K* represents the number of intermediate subpopulations in the 1D linear admixture data (left panel), and the number of independent subpopulations in the independent data (right panel)

Results for selection of important predictors, as measured by the mean TPR in 50 replications, are presented in Figure 1. For both 1D linear admixture and independent subpopulations, glmnet without PC adjustment failed to retrieve causal markers compared to all other methods. This is expected under population stratification; SNPs that differ in frequency between subpopulations are identified as important predictors because prevalence is not constant across each group. When the first 10 PCs were added as unpenalized covariates, glmnetPC’s ability to select causal predictors was lesser to that of pglmm and ggmix for the 20 independent subpopulations. Since in the independent subpopulations simulated data, each subpopulation indicator function is strongly associated with only a few PCs, as shown in Supplementary Appendix SE, omitting to include all important PCs in the model leads to incorrectly capturing the confounding structure. On the other hand, because there is more overlap between subpopulations in the admixture data compared to the independent subpopulations (Reisetter and Breheny, 2021), each subpopulation indicator function is moderately correlated with many PCs. Thus, including only the first 10 PCs in the model is enough to correct for confounding even when *K* = 20 (bottom-left panel of Fig. 1). Alternatively, including a random effect with variance–covariance structure proportional to the GSM correctly adjusts for population structure in all scenarios while alleviating the burden of choosing the right number of fixed predictors to include in the model. Even though ggmix assumes a standard LMM for the binary trait, it was able to identify causal markers at the same rate as pglmm.

**Fig. 2.**
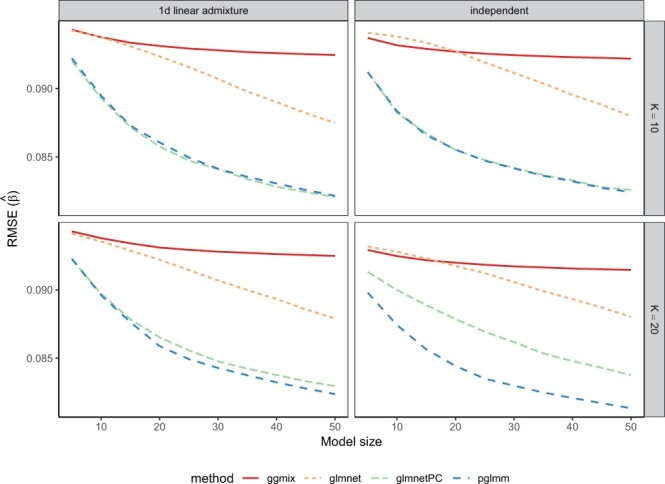
Mean of 50 RMSEs for the first simulation scenario where we simulated random genotypes from the BN-PSD admixture model. *K* represents the number of intermediate subpopulations in the 1D linear admixture data (left panel), and the number of independent subpopulations in the independent data (right panel)

Results for estimation of SNP effects as measured by the mean RMSE in 50 replications are presented in Figure 2. Results are consistent with TPR results in that glmnet without PC adjustment performed poorly in all scenarios, while pglmm outperformed all other methods for the 20 independent subpopulations and performed comparably with glmnetPC for all other settings. As expected, ggmix had higher RMSE compared to pglmm and glmnetPC. Thus, even though ggmix was able to identify causal markers at the same rate as other methods that accounted for the binary nature of the response, resulting estimates for the SNP effects were not accurate.

**Fig. 3.**
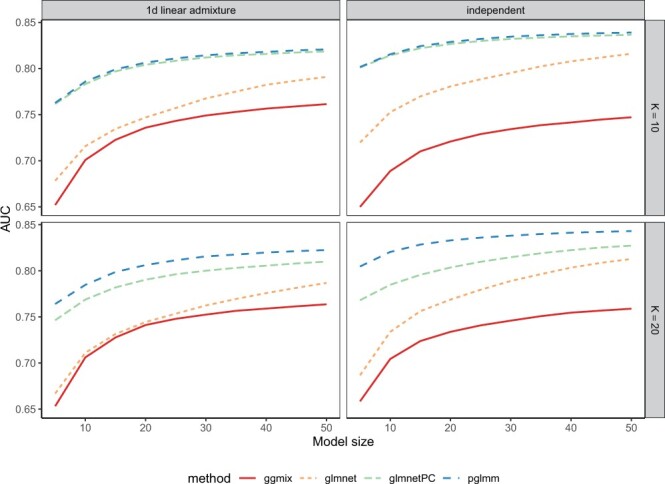
Mean of 50 AUCs in test sets for the first simulation scenario where we simulated random genotypes from the BN-PSD admixture model. *K* represents the number of intermediate subpopulations in the 1D linear admixture data (left panel), and the number of independent subpopulations in the independent data (right panel)

For both 1D linear admixture and independent subpopulations, ggmix and glmnet had poor predictive performance for *K *=* *10 and *K *=* *20, as reported in Figure 3. Also, the predictive performance of glmnetPC was greatly reduced when *K *=* *20 for both admixture and independent populations, even if in the case of the admixture data, the RMSE for estimation of SNP effects was comparable for glmnetPC and pglmm. This suggests that the observed discrepancy in predictive accuracy might be caused by how each method handle the confounding effects. Using only 10 PCs as fixed effects when *K *=* *20 may result in overfitted coefficients for glmnetPC, which may in turn potentially decrease prediction accuracy and increase variance of predictions in independent subjects. By using a ridge-like estimator for the random effects, pglmm is less likely to overfit the confounding effects compared to glmnetPC.

### 3.2 Simulation results for the second scenario

In the second simulation scenario, we evaluated the performance of our method when using AIC or cross-validation as a model selection strategy, i.e. for selecting the optimal value of the regularization parameter, rather than fixing the number of active predictors in the model. For all other methods, we used cross-validation to perform model selection and compared the ability of all methods to adjust for potential confounding stemming from subjects’ relatedness. We present median and interquartile values for AUC, model size, RMSE, TPR and precision in Table 3. In addition to the penalized methods, we reported prediction accuracy for the standard gBLUP model where only non-genetic covariates and a polygenic random effect were included.

**Table 3.** Results of the model selection simulations for the second scenario

	ggmix	glmnet	glmnetPC	pglmm (AIC)	pglmm (CV)	gBLUP
Model size	341 (1598)	226 (1050)	378 (1021)	50.5 (54.2)	102 (487)	0 (0)
AUC	0.558 (0.023)	0.561 (0.030)	0.552 (0.022)	0.569 (0.021)	0.568 (0.028)	0.549 (0.020)
RMSE	0.0334 (0.0038)	0.0328 (0.0117)	0.0336 (0.0127)	0.0318 (0.0036)	0.0324 (0.0051)	–
TPR	0.167 (0.248)	0.133 (0.192)	0.17 (0.165)	0.06 (0.0517)	0.08 (0.155)	–
Precision	0.0576 (0.141)	0.0854 (0.117)	0.0584 (0.0653)	0.203 (0.156)	0.107 (0.200)	–

*Note*: For each replication, the best model for pglmm was chosen using either AIC, or CV. For all other methods, the best model was chosen using CV. For all metrics, we report median and interquartile range. Since the gBLUP model makes prediction using only non-genetic covariates and a polygenic random effect, we only report median AUC values.

Contrarily to the previous simulations under the admixture and independent populations models, glmnetPC had lower prediction accuracy compared to glmnet. This highlights the fact that using a fixed number of PCs to control for sample relatedness is not robust compared to using a random effect. In comparison to the first simulation scenario, where the TPR was between 30% and 40% when the number of active predictors in the model was equal to the number of causal markers, the maximum value for the TPR for all methods was equal to 17% in the second simulation scenario. This is because although in both scenarios the proportion of causal markers was the same (c=1%), we simulated more causal predictors with weaker effects size in the second scenario. Indeed, the number of causal markers and simulated heritability in the second scenario were equal to c*p=150 and $h_{g}^{2}=17\%$, respectively, compared to c*p=50 and $h_{g}^{2}=50\%$ in the first scenario.

In term of prediction accuracy and estimation of predictor coefficients, pglmm performed comparably using either cross-validation or AIC, while achieving better performance than all other methods. Moreover, our method led to sparser models with higher precision than all other methods, especially when using AIC as a model selection criteria. Thus, using a logistic lasso model with 10 PCs to control for relatedness led to models with more false positives and worse prediction accuracy than all other methods, including the logistic lasso with no PC adjustment. These results highlight once again the robustness of using a random effect rather than PCs to account for relatedness between subjects. In summary, by explicitly modeling the correlation between subjects and binary nature of the trait, our method led to sparser models with higher precision and prediction accuracy than all other methods.

### 3.3 PRS for the UK Biobank real data application

Results for asthma and high cholesterol PRSs are summarized in Table 4. For asthma, pglmm performed better than all other methods when comparing AUC on the test sets. In addition, the median number of predictors selected by pglmm was 2.5 times smaller than for glmnetPC, and the variability in predictors selected was more important for glmnetPC, as reported by an IQR value equal to 113.25, compared to 43.25 for pglmm. This is consistent with our simulation results showing that pglmm leads to sparser models with higher predictive power than logistic lasso. For high cholesterol, the median number of predictors selected by both penalized models was equal or close to 0, which suggests that SNP effects may be too small to detect. Indeed, both methods based on sparse regression do not perform as well as either the gBLUP or null model with non-genetic covariates and 10 PCs. For both asthma and high cholesterol, fitting the null model for pglmm took a median time of approximately 1.7 min, while fitting the full lasso path for 100 values of the tuning parameter *λ* took a median time of 51 and 55 min, respectively. Analyses were performed using 2 cores of an AMD Rome 7532 (2.40 GHz), each with 64GB of RAM. As implemented in the glmnet package and other high-dimensional sparse regression methods, we use sequential strong rules for solving the lasso problem such that most of the predictors are discarded from the optimization problem at each iteration (Tibshirani *et al.*, 2011). This allows our sparse regularized mixed regression method to remain computationally efficient when the number of genetic variants is very large.

**Table 4.** PRS results for asthma and high cholesterol using a total of 6731 subjects of White British ancestry from the UK Biobank data having estimated first, second or third degree relationships with at least one other individual

Model	AUC_test_	Model size
Asthma	Mean (SD)	Median (IQR)
Covariates + 10PCs	0.5227 (0.021)	–
gBLUP	0.5447 (0.017)	–
glmnetPC	0.5258 (0.020)	42 (113.25)
pglmm	0.5484 (0.017)	16.5 (43.25)
High cholesterol		
Covariates + 10PCs	0.7126 (0.020)	–
gBLUP	0.7142 (0.020)	–
glmnetPC	0.7106 (0.020)	0 (7.25)
pglmm	0.7118 (0.020)	0.5 (16)

*Note*: To find the optimal regularization parameter for both penalized methods, we split the subjects in training (80%) and test (20%) sets for a total of 40 times.

### 3.4 Computational efficiency

In this additional simulation scenario, we compared the computational efficiency of all methods. We considered a grid of values for the sample size *n* and number of predictors *p*, and we simulated a total of 10 replications of the 1D linear admixture model with 20 populations, for each of the nine combinations of (*n*, *p*). For each replication, we randomly selected 1% of the predictors to be causal. Simulations were performed on a single core of an AMD Rome 7532 (2.40 GHz) with 64 GB of RAM. Results for the computational efficiency of all methods for different sample sizes and number of predictors are reported in Table 5. The median computational time of pglmm ranged between 5.3 min (*n *=* *2500, p=10 000) and 48.6 min (*n *=* *7500, p=30 000), while ggmix running time varied between 13.7 and 93.3 min, respectively. Thus, pglmm was considerably faster than ggmix because contrarily to the latter, we estimate the variance components only once under the null model, which dramatically decreases the computational complexity of the regularized minimization problem. The maximum running time for glmnetPC was equal to 2 min, for the simulations with *n *=* *7500 and p=30 000. The large difference in computation time between pglmm and glmnetPC is explained by the dimension of the parameter space that each method is estimating. Indeed, to account for population structure, glmnetPC only needs to fit the first 10 PCs, while under the mixed model approach, pglmm needs to estimate the random effects vector of dimension equal to the sample size. As we show in Supplementary Appendix SB, by profiling out the random effects vector from the regularized minimization problem in our proposed algorithm, we need to rotate the response vector using the eigenvectors of the variance–covariance matrix after each WLS iteration such that the transformed data is uncorrelated. This requires performing multiple matrix-vector multiplications, with complexity $O(n^{2})$, while glmnetPC only needs performing vector multiplications with complexity *O*(*n*).

**Table 5.** Median computation time in minutes of pglmm, glmnetPC and ggmix for fitting a sequence of 100 regression models for different sample sizes and number of predictors

*n*	*p*	pglmm		glmnetPC	ggmix
		Null model	Full model	Full model	Full model
2500	10 000	0.6	5.3	0.2	13.7
	20 000	–	11.0	0.4	26.6
	30 000	–	13.8	0.6	34.0
5000	10 000	2.1	11.9	0.6	25.0
	20 000	–	24.4	1.0	42.0
	30 000	–	34.9	1.2	55.8
7500	10 000	4.8	24.0	1.3	51.3
	20 000	–	33.5	1.7	78.2
	30 000	–	48.6	2.0	93.3

*Note*: For pglmm, we also present the median computation time for fitting the null model. Simulations were performed on a single core of an AMD Rome 7532 (2.40 GHz) with 64 GB of RAM. We simulated a total of 10 replications of the 1D linear admixture model with 20 populations.

## 4 Discussion

We have introduced a new method called pglmm based on regularized PQL estimation, for selecting important predictors and estimating their effects in high-dimensional GWAS data, accounting for population structure, close relatedness and binary nature of the trait. By simulating random genotypes from the BN-PSD admixture model for 10 or 20 subpopulations with 1D geography or independent subpopulations, we showed that pglmm was markedly better than a logistic lasso with PC adjustment when the number of subpopulations was greater than the number of PCs included. We also showed that a lasso LMM was unable to estimate predictor effects with accuracy for binary responses, which greatly decreased its predictive performance. Performance assessment was based on TPR of selected predictors, RMSE of estimated effects and AUC of predictions. These results strongly advocate for using methods that explicitly account for the binary nature of the trait while effectively controlling for population structure and relatedness in genetic studies.

In the second simulation scenario, we used real genotype data from a subset of related individuals from the UK Biobank data to simulate binary responses, and showed that pglmm effectively led to sparser models with higher precision and prediction accuracy than a lasso LMM and a logistic lasso model with or without PC adjustment. We also demonstrated that using AIC as a model selection strategy led to similar prediction performance than cross-validation, with even sparser models. Using the same dataset, we illustrated the potential advantages of pglmm over a logistic lasso with PC adjustment in a real data application for constructing a PRS on two highly heritable binary traits. Although these analyses have limited power compared to using all UK Biobank subjects of White British ancestry, it is often the case that researchers may be limited to relatively smaller datasets. For these cases, it is of primary importance to avoid discarding samples based on relatedness and properly account for the possible correlation between observations. Thus, we used this reduced sample from the UK Biobank to demonstrate the potential advantages of using penalized multivariable GLMMs in smaller datasets where subjects’ relatedness might be an important confounder.

A limitation of pglmm compared to a logistic lasso with PC adjustment is the computational cost of performing multiple matrix calculations that comes from incorporating a GSM to account for population structure and relatedness between individuals. These computations are clearly too prohibitive for application to large cohorts such as the full UK Biobank with a total of 500*K* samples. Solutions to explore in order to increase computation speed and decrease memory usage would be the use of conjugate gradient methods with a diagonal preconditioner matrix, as proposed by Zhou *et al.* (2018), and to use a sparse GSM to adjust for the sample relatedness (Jiang *et al.*, 2019).

In this study, we focused solely on the lasso as a regularization penalty for the genetic markers effects. However, it is known that estimated effects by lasso will have large biases because the resulting shrinkage is constant irrespective of the magnitude of the effects. Alternative regularizations like the Smoothly Clipped Absolute Deviation (SCAD) (Fan and Li, 2001) and Minimax Concave Penalty (MCP) (Zhang, 2010) could be explored, although we note that both SCAD and MCP require tuning an additional parameter which controls the relaxation rate of the regularization. Another alternative includes implementation of the relaxed lasso, which has shown to produce sparser models with equal or lower prediction loss than the regular lasso estimator for high-dimensional data (Meinshausen, 2007). Finally, it would also be of interest to explore if tuning the generalized ridge regularization on the random effects, or replacing it by a lasso regularization to perform selection of individual random effects, could result in better predictive performance.

## Supplementary Material

btad063_Supplementary_Data

## Data Availability

Our Julia package PenalizedGLMM and simulated data are available on github https://github.com/julstpierre/PenalizedGLMM. UK Biobank data are available via application directly to UK Biobank (https://www.ukbiobank.ac.uk/enable-your-research). The current study was conducted under UK Biobank application number 20802.
