# Supplementary material for: Efficient penalized generalized linear mixed models for variable selection and genetic risk prediction in high-dimensional data
Source: Bioinformatics. 2023 Jan 27;39(2):btad063. doi: 10.1093/bioinformatics/btad063 (PMC9907224; doi:10.1093/bioinformatics/btad063)
Supplement: btad063_Supplementary_Data [file btad063_supplementary_data.pdf]

Supplementary Material to Efficient Penalized Generalized Linear Mixed Models for Variable Selection and Genetic Risk Prediction in High-Dimensional Data

JULIEN ST-PIERRE*

Department of Epidemiology, Biostatistics and Occupational Health,

McGill University, Montreal, Quebec, Canada

julien.st-pierre@mail.mcgill.ca

KARIM OUALKACHA

Département de Mathématiques,

Université du Québec à Montréal, Montreal, Quebec, Canada

SAHIR RAI BHATNAGAR

Department of Epidemiology, Biostatistics and Occupational Health,

McGill University, Montreal, Quebec, Canada

January 24, 2023

*To whom correspondence should be addressed.

Appendix A. Estimation of Variance Component Parameters

In what follows, we provide a near verbatim of Appendix A from Chen et al. (2016) that details the AI-REML algorithm to estimate the variance components and fixed effects for the non-genetic covariates under the assumption of no genetic association. To be consistent with the remainder of the manuscript, we slightly changed the notation from the original derivation where appropriate.

If ϕ and $\boldsymbol{\tau}$ are known, we jointly choose $\hat{\boldsymbol{\alpha}}(\phi, \boldsymbol{\tau})$, $\hat{\boldsymbol{\gamma}}(\phi, \boldsymbol{\tau})$ and $\hat{\boldsymbol{b}}(\phi, \boldsymbol{\tau})$ to minimize (2), then $\hat{\boldsymbol{b}}(\phi, \boldsymbol{\tau}) = \tilde{\boldsymbol{b}}(\hat{\boldsymbol{\alpha}}(\phi, \boldsymbol{\tau}), \hat{\gamma}(\phi, \boldsymbol{\tau}))$ because $\tilde{\boldsymbol{b}}$ maximizes $f(\boldsymbol{b})$ for given $(\boldsymbol{\alpha}, \gamma)$. Assuming that the weights in $\boldsymbol{W}$ vary slowly with the conditional mean, the derivatives of (2) at $\boldsymbol{\gamma} = 0$ with respect to $(\boldsymbol{\alpha}, \boldsymbol{b})$ are given by

$$\frac{\partial ql(\boldsymbol{\alpha}, \boldsymbol{\gamma} = 0, \phi, \boldsymbol{\tau})}{\partial \boldsymbol{\alpha}} = -\sum_{i=1}^{n} \frac{a_i(y_i - \mu_i)}{\phi \nu(\mu_i)} \frac{1}{g'(\mu_i)} \boldsymbol{X}_i^\intercal = -\boldsymbol{X}^\intercal \boldsymbol{W} \Delta (\boldsymbol{y} - \boldsymbol{\mu}),$$

$$\frac{\partial ql(\boldsymbol{\alpha}, \boldsymbol{\gamma} = 0, \phi, \boldsymbol{\tau})}{\partial \boldsymbol{b}} = -\sum_{i=1}^{n} \frac{a_i(y_i - \mu_i)}{\phi \nu(\mu_i)} \frac{1}{g'(\mu_i)} \boldsymbol{Z}_i^\intercal + \left(\sum_{s=1}^{S} \tau_s \boldsymbol{V}_s\right)^{-1} \boldsymbol{b} = \left(\sum_{s=1}^{S} \tau_s \boldsymbol{V}_s\right)^{-1} \boldsymbol{b} - \boldsymbol{W} \Delta (\boldsymbol{y} - \boldsymbol{\mu}),$$

where $\Delta = \mathrm{diag}(g'(\mu_i))$ and $\boldsymbol{Z}_i$ is a $n \times 1$ vector of indicators such that $b_i = \boldsymbol{Z}_i \boldsymbol{b}$. Defining the working vector $\tilde{\boldsymbol{Y}}$ with elements $\tilde{Y}_i = \eta_i + g'(\mu_i)(y_i - \mu_i)$, the solution of

$$\begin{cases} \boldsymbol{X}^\intercal \boldsymbol{W} \Delta (\boldsymbol{y} - \boldsymbol{\mu}) = 0 \\ \boldsymbol{W} \Delta (\boldsymbol{y} - \boldsymbol{\mu}) = \left(\sum_{s=1}^{S} \tau_s \boldsymbol{V}_s\right)^{-1} \boldsymbol{b} \end{cases}$$

can be written as the solution to the system

$$\begin{bmatrix} \boldsymbol{X}^\intercal \boldsymbol{W} \boldsymbol{X} & \boldsymbol{X}^\intercal \boldsymbol{W} \\ \boldsymbol{W} \boldsymbol{X} & \left(\sum_{s=1}^{S} \tau_s \boldsymbol{V}_s\right)^{-1} + \boldsymbol{W} \end{bmatrix} \begin{bmatrix} \boldsymbol{\alpha} \\ \boldsymbol{b} \end{bmatrix} = \begin{bmatrix} \boldsymbol{X}^\intercal \boldsymbol{W} \tilde{\boldsymbol{Y}} \\ \boldsymbol{W} \tilde{\boldsymbol{Y}} \end{bmatrix}.$$

Let $\boldsymbol{\Sigma} = \boldsymbol{W}^{-1} + \sum_{s=1}^{S} \tau_s \boldsymbol{V}_s$, $\boldsymbol{P} = \boldsymbol{\Sigma}^{-1} - \boldsymbol{\Sigma}^{-1} \boldsymbol{X} \left(\boldsymbol{X}^\intercal \boldsymbol{\Sigma}^{-1} \boldsymbol{X}\right)^{-1} \boldsymbol{X}^\intercal \boldsymbol{\Sigma}^{-1}$, then

$$\begin{cases} \hat{\boldsymbol{\alpha}} = \left(\boldsymbol{X}^\intercal \boldsymbol{\Sigma}^{-1} \boldsymbol{X}\right)^{-1} \boldsymbol{X}^\intercal \boldsymbol{\Sigma}^{-1} \tilde{\boldsymbol{Y}} \\ \hat{\boldsymbol{b}} = \left(\sum_{s=1}^{S} \tau_s \boldsymbol{V}_s\right) \boldsymbol{\Sigma}^{-1} \left(\tilde{\boldsymbol{Y}} - \boldsymbol{X} \hat{\boldsymbol{\alpha}}\right) \end{cases}.$$

Of note, we have that

$$\begin{aligned} \tilde{\boldsymbol{Y}} - \hat{\boldsymbol{\eta}} &= \tilde{\boldsymbol{Y}} - \boldsymbol{X} \hat{\boldsymbol{\alpha}} - \hat{\boldsymbol{b}} \\ &= \left\{ \boldsymbol{I} - \left(\sum_{s=1}^{S} \tau_s \boldsymbol{V}_s\right) \boldsymbol{\Sigma}^{-1} \right\} \left(\tilde{\boldsymbol{Y}} - \boldsymbol{X} \hat{\boldsymbol{\alpha}}\right) \\ &= \boldsymbol{W}^{-1} \boldsymbol{\Sigma}^{-1} \left(\tilde{\boldsymbol{Y}} - \boldsymbol{X} \hat{\boldsymbol{\alpha}}\right) \\ &= \boldsymbol{W}^{-1} \boldsymbol{P} \tilde{\boldsymbol{Y}}. \end{aligned}$$

The log integrated quasi-likelihood function in (2) evaluated at $(\hat{\boldsymbol{\alpha}}, \boldsymbol{\gamma} = 0, \phi, \boldsymbol{\tau})$ becomes

$$\begin{aligned}
ql(\hat{\boldsymbol{\alpha}}(\phi, \boldsymbol{\tau}), \boldsymbol{\gamma} = 0, \phi, \boldsymbol{\tau}) &= -\frac{1}{2}\log\left|\sum_{s=1}^{S} \tau_s \boldsymbol{V}_s \boldsymbol{W} + \boldsymbol{I}\right| - \frac{1}{2}\sum_{i=1}^{n} \frac{a_i(y_i - \hat{\mu}_i)^2}{\phi\nu(\hat{\mu}_i)} - \frac{1}{2}\hat{\boldsymbol{b}}^{\intercal}\left(\sum_{s=1}^{S} \tau_s \boldsymbol{V}_s\right)\hat{\boldsymbol{b}} \\
&= -\frac{1}{2}\log|\boldsymbol{\Sigma}\boldsymbol{W}| - \frac{1}{2}(\tilde{\boldsymbol{Y}} - \hat{\boldsymbol{\eta}})^{\intercal}\boldsymbol{W}(\tilde{\boldsymbol{Y}} - \hat{\boldsymbol{\eta}}) \\
&\quad - \frac{1}{2}\left(\tilde{\boldsymbol{Y}} - \boldsymbol{X}\hat{\boldsymbol{\alpha}}\right)^{\intercal}\boldsymbol{\Sigma}^{-1}\left(\sum_{s=1}^{S} \tau_s \boldsymbol{V}_s\right)\boldsymbol{\Sigma}^{-1}\left(\tilde{\boldsymbol{Y}} - \boldsymbol{X}\hat{\boldsymbol{\alpha}}\right) \\
&= -\frac{1}{2}\log|\boldsymbol{W}| - \frac{1}{2}\log|\boldsymbol{\Sigma}| - \frac{1}{2}\tilde{\boldsymbol{Y}}^{\intercal}\boldsymbol{P}\boldsymbol{W}^{-1}\boldsymbol{P}\tilde{\boldsymbol{Y}} \\
&\quad - \frac{1}{2}\tilde{\boldsymbol{Y}}^{\intercal}\boldsymbol{P}\left(\sum_{s=1}^{S} \tau_s \boldsymbol{V}_s\right)\boldsymbol{P}\tilde{\boldsymbol{Y}} \\
&\quad = c - \frac{1}{2}\log|\boldsymbol{\Sigma}| - \frac{1}{2}\tilde{\boldsymbol{Y}}^{\intercal}\boldsymbol{P}\boldsymbol{\Sigma}\boldsymbol{P}\tilde{\boldsymbol{Y}} \\
&\quad = c - \frac{1}{2}\log|\boldsymbol{\Sigma}| - \frac{1}{2}\tilde{\boldsymbol{Y}}^{\intercal}\boldsymbol{P}\tilde{\boldsymbol{Y}}.
\end{aligned}$$

Similarly, the restricted maximum likelihood (REML) version is

$$ql_R(\hat{\boldsymbol{\alpha}}(\phi, \boldsymbol{\tau}), \boldsymbol{\gamma} = 0, \phi, \boldsymbol{\tau}) = c_R - \frac{1}{2}\log|\boldsymbol{\Sigma}| - \frac{1}{2}\log\left|\boldsymbol{X}^{\intercal}\boldsymbol{\Sigma}^{-1}\boldsymbol{X}\right| - \frac{1}{2}\tilde{\boldsymbol{Y}}^{\intercal}\boldsymbol{P}\tilde{\boldsymbol{Y}}.$$

We need to maximize $ql_R(\hat{\boldsymbol{\alpha}}(\phi, \boldsymbol{\tau}), \boldsymbol{\gamma} = 0, \phi, \boldsymbol{\tau})$ with respect to $\phi, \boldsymbol{\tau}$. Let $\boldsymbol{V}_0 = \text{diag}\{a_i^{-1}\nu(\mu_i)[g'(\mu_i)^2]\} = \phi^{-1}\boldsymbol{W}^{-1}$, then $\boldsymbol{\Sigma} = \phi\boldsymbol{V}_0 + \sum_{s=1}^{S} \tau_s \boldsymbol{V}_s$, and the first derivatives of $ql_R(\hat{\boldsymbol{\alpha}}(\phi, \boldsymbol{\tau}), \boldsymbol{\gamma} = 0, \phi, \boldsymbol{\tau})$ with respect to ϕ and τ_s are

$$\frac{\partial ql_R(\hat{\boldsymbol{\alpha}}(\phi, \boldsymbol{\tau}), \boldsymbol{\gamma} = 0, \phi, \boldsymbol{\tau})}{\partial \phi} = \frac{1}{2}\left\{\tilde{\boldsymbol{Y}}^{\intercal}\boldsymbol{P}\boldsymbol{V}_0\boldsymbol{P}\tilde{\boldsymbol{Y}} - tr(\boldsymbol{P}\boldsymbol{V}_0)\right\} \tag{9}$$

$$\frac{\partial ql_R(\hat{\boldsymbol{\alpha}}(\phi, \boldsymbol{\tau}), \boldsymbol{\gamma} = 0, \phi, \boldsymbol{\tau})}{\partial \tau_s} = \frac{1}{2}\left\{\tilde{\boldsymbol{Y}}^{\intercal}\boldsymbol{P}\boldsymbol{V}_s\boldsymbol{P}\tilde{\boldsymbol{Y}} - tr(\boldsymbol{P}\boldsymbol{V}_s)\right\}, \tag{10}$$

since one can show that

$$\frac{\partial \boldsymbol{P}}{\partial \phi} = -\boldsymbol{P}\boldsymbol{V}_0\boldsymbol{P}, \qquad \frac{\partial \boldsymbol{P}}{\partial \tau_s} = -\boldsymbol{P}\boldsymbol{V}_s\boldsymbol{P}.$$

$\hat{\phi}$ and $\hat{\boldsymbol{\tau}}$ are estimated by finding the solutions of (9) and (10) equal to zero. Let $\boldsymbol{\theta} = (\phi, \boldsymbol{\tau})$, and recall that in the REML iterative process, $\hat{\boldsymbol{\theta}}$ at the (i+1)th iteraton is updated by $\hat{\boldsymbol{\theta}}^{(i+1)} = \hat{\boldsymbol{\theta}}^{(i)} + J(\hat{\boldsymbol{\theta}}^{(i)})^{-1}S(\hat{\boldsymbol{\theta}}^{(i)})$, where $S(\boldsymbol{\theta}) = \frac{\partial ql_R(\boldsymbol{\theta})}{\partial \boldsymbol{\theta}}$ and $J(\boldsymbol{\theta}) = -\frac{\partial^2 ql_R(\boldsymbol{\theta})}{\partial \boldsymbol{\theta}^2}$. The elements of the observed information matrix $J(\boldsymbol{\theta})$

are

$$
\begin{aligned}
-\frac{\partial^2 ql_R(\hat{\boldsymbol{\alpha}}(\phi,\boldsymbol{\tau}),\gamma=0,\phi,\boldsymbol{\tau})}{\partial\phi^2} &= \tilde{\boldsymbol{Y}}^\intercal \boldsymbol{P}\boldsymbol{V}_0\boldsymbol{P}\boldsymbol{V}_0\boldsymbol{P}\tilde{\boldsymbol{Y}} - \frac{1}{2}tr(\boldsymbol{P}\boldsymbol{V}_0\boldsymbol{P}\boldsymbol{V}_0)\\
-\frac{\partial^2 ql_R(\hat{\boldsymbol{\alpha}}(\phi,\boldsymbol{\tau}),\gamma=0,\phi,\boldsymbol{\tau})}{\partial\phi\partial\tau_s} &= \tilde{\boldsymbol{Y}}^\intercal \boldsymbol{P}\boldsymbol{V}_0\boldsymbol{P}\boldsymbol{V}_s\boldsymbol{P}\tilde{\boldsymbol{Y}} - \frac{1}{2}tr(\boldsymbol{P}\boldsymbol{V}_0\boldsymbol{P}\boldsymbol{V}_s)\\
-\frac{\partial^2 ql_R(\hat{\boldsymbol{\alpha}}(\phi,\boldsymbol{\tau}),\gamma=0,\phi,\boldsymbol{\tau})}{\partial\tau_l\partial\tau_s} &= \tilde{\boldsymbol{Y}}^\intercal \boldsymbol{P}\boldsymbol{V}_l\boldsymbol{P}\boldsymbol{V}_s\boldsymbol{P}\tilde{\boldsymbol{Y}} - \frac{1}{2}tr(\boldsymbol{P}\boldsymbol{V}_l\boldsymbol{P}\boldsymbol{V}_s).
\end{aligned}
$$

The elements of the expected information matrix are

$$
\begin{aligned}
E\left(-\frac{\partial^2 ql_R(\hat{\boldsymbol{\alpha}}(\phi,\boldsymbol{\tau}),\gamma=0,\phi,\boldsymbol{\tau})}{\partial\phi^2}\right) &= \frac{1}{2}tr(\boldsymbol{P}\boldsymbol{V}_0\boldsymbol{P}\boldsymbol{V}_0)\\
E\left(-\frac{\partial^2 ql_R(\hat{\boldsymbol{\alpha}}(\phi,\boldsymbol{\tau}),\gamma=0,\phi,\boldsymbol{\tau})}{\partial\phi\partial\tau_s}\right) &= \frac{1}{2}tr(\boldsymbol{P}\boldsymbol{V}_0\boldsymbol{P}\boldsymbol{V}_s)\\
E\left(-\frac{\partial^2 ql_R(\hat{\boldsymbol{\alpha}}(\phi,\boldsymbol{\tau}),\gamma=0,\phi,\boldsymbol{\tau})}{\partial\tau_l\partial\tau_s}\right) &= \frac{1}{2}tr(\boldsymbol{P}\boldsymbol{V}_l\boldsymbol{P}\boldsymbol{V}_s).
\end{aligned}
$$

The average information matrix $\boldsymbol{AI}$ is defined as the average of the observed information $J(\boldsymbol{\theta})$ and the expected information

$$
\begin{aligned}
\boldsymbol{AI}_{\phi\phi} &= \frac{1}{2}\tilde{\boldsymbol{Y}}^\intercal \boldsymbol{P}\boldsymbol{V}_0\boldsymbol{P}\boldsymbol{V}_0\boldsymbol{P}\tilde{\boldsymbol{Y}},\\
\boldsymbol{AI}_{\phi\tau_s} &= \frac{1}{2}\tilde{\boldsymbol{Y}}^\intercal \boldsymbol{P}\boldsymbol{V}_0\boldsymbol{P}\boldsymbol{V}_s\boldsymbol{P}\tilde{\boldsymbol{Y}},\\
\boldsymbol{AI}_{\tau_s\tau_l} &= \frac{1}{2}\tilde{\boldsymbol{Y}}^\intercal \boldsymbol{P}\boldsymbol{V}_s\boldsymbol{P}\boldsymbol{V}_l\boldsymbol{P}\tilde{\boldsymbol{Y}}.
\end{aligned}
$$

Let $\boldsymbol{\theta}$ be the variance component and dispersion parameters to estimate, that is when $\phi \neq 1$, $\boldsymbol{\theta} = (\phi, \boldsymbol{\tau})$, and $\boldsymbol{AI}$ is a $(S+1)\times(S+1)$ matrix. For binary data, $\phi = 1$, $\boldsymbol{\theta} = \boldsymbol{\tau}$, and $\boldsymbol{AI}$ is a $S \times S$ matrix containing only $\boldsymbol{AI}_{\tau_s\tau_l}$. We use the following algorithm to estimate $\boldsymbol{\theta}$, $\boldsymbol{\alpha}$ and $\boldsymbol{b}$:

Algorithm 1: AI-REML algorithm

1. *Initialization*

   Fit a generalized linear model with $\boldsymbol{\tau} = 0$ and get $\hat{\boldsymbol{\alpha}}^{(0)}$ and working vector $\tilde{\boldsymbol{Y}}^{(0)}$;

   Use $\boldsymbol{\theta}^{(0)} = Var(\tilde{\boldsymbol{Y}}^{(0)})/S$ (if $\phi = 1$) or $\boldsymbol{\theta}^{(0)} = Var(\tilde{\boldsymbol{Y}}^{(0)})/(S+1)$ (if $\phi \neq 1$) as the initial value of $\boldsymbol{\theta}$;

   For each $s = 0, 1, ..., S,$ update $\boldsymbol{\theta}$ using $\theta_s^{(1)} = \theta_s^{(0)} + 2n^{-1}\{\theta_s^{(0)}\}^2(\partial ql_R(\boldsymbol{\theta}^{(0)})/\partial\theta_s)$;

2. *Iteration*

   for $t = 1, 2, ...,$ until convergence **do**

   Update $\boldsymbol{\theta}^{(t+1)} = \boldsymbol{\theta}^{(t)} + \{\boldsymbol{AI}^{(t)}\}^{-1}(\partial ql_R(\boldsymbol{\theta}^{(t)})/\partial\boldsymbol{\theta})$;

   Calculate $\hat{\boldsymbol{\alpha}}^{(t+1)}$ and $\hat{\boldsymbol{b}}^{(t+1)}$ using $\tilde{\boldsymbol{Y}}^{(t)}$ and $\boldsymbol{\theta}^{(t+1)}$;

   Update $\tilde{\boldsymbol{Y}}^{(t+1)}$ using $\hat{\boldsymbol{\alpha}}^{(t+1)}$ and $\hat{\boldsymbol{b}}^{(t+1)}$;

Convergence is defined using

$2\max\{|\hat{\boldsymbol{\alpha}}^{(t)} - \hat{\boldsymbol{\alpha}}^{(t-1)}|/(|\hat{\boldsymbol{\alpha}}^{(t)}| + |\hat{\boldsymbol{\alpha}}^{(t-1)}|), |\hat{\boldsymbol{\theta}}^{(t)} - \hat{\boldsymbol{\theta}}^{(t-1)}|/(|\hat{\boldsymbol{\theta}}^{(t)}| + |\hat{\boldsymbol{\theta}}^{(t-1)}|)\} \leq$ tolerance

Appendix B. Cyclic coordinate Descent for PQL Regularized Parameters

Assuming that the variance components and dispersion parameters are known, we fit the full GLMM (1) with lasso regularization on $\boldsymbol{\beta} = (\boldsymbol{\alpha}^\intercal, \boldsymbol{\gamma}^\intercal)^\intercal$ to obtain PQL regularized estimates for $\boldsymbol{\beta}$ and $\tilde{\boldsymbol{b}}$. At each iteration, we cycle through the coordinates and minimize the objective function (3) with respect to one coordinate only. Suppose we have estimates $\tilde{\boldsymbol{\beta}}$ and we wish to partially optimize (3) with respect to $\tilde{\boldsymbol{b}}$. The gradient and Hessian of ℓ_{PQL} with respect to $\tilde{\boldsymbol{b}}$ at $\boldsymbol{\beta} = \tilde{\boldsymbol{\beta}}$ are given by

$$\begin{aligned}\nabla_{\tilde{\boldsymbol{b}}}\ell_{PQL}(\tilde{\boldsymbol{\beta}}, \hat{\phi}, \hat{\boldsymbol{\tau}}|\tilde{\boldsymbol{b}}) &= \sum_{i=1}^{n} \frac{a_i(y_i - \mu_i)}{\hat{\phi}\nu(\mu_i)} \frac{1}{g'(\mu_i)} \boldsymbol{Z}_i^\intercal - \left(\sum_{s=1}^{S} \hat{\tau}_s \boldsymbol{V}_s\right)^{-1} \tilde{\boldsymbol{b}} \\ &= \boldsymbol{W}\Delta(\boldsymbol{y} - \boldsymbol{\mu}) - \left(\sum_{s=1}^{S} \hat{\tau}_s \boldsymbol{V}_s\right)^{-1} \tilde{\boldsymbol{b}},\end{aligned}$$

and

$$\nabla^2_{\tilde{\boldsymbol{b}}}\ell_{PQL}(\tilde{\boldsymbol{\beta}}, \hat{\phi}, \hat{\boldsymbol{\tau}}|\tilde{\boldsymbol{b}}) = -\boldsymbol{W} - \left(\sum_{s=1}^{S} \hat{\tau}_s \boldsymbol{V}_s\right)^{-1},$$

where $\Delta = \text{diag}(g'(\mu_i))$ and $\boldsymbol{Z}_i$ is a $n \times 1$ vector of indicators such that $b_i = \boldsymbol{Z}_i\boldsymbol{b}$. We form a quadratic approximation of $\ell_{PQL}(\tilde{\boldsymbol{\beta}}, \hat{\phi}, \hat{\boldsymbol{\tau}}|\tilde{\boldsymbol{b}})$ around current iterate $\tilde{\boldsymbol{b}}$, which yields

$$f(\boldsymbol{b}) := \ell_{PQL}(\tilde{\boldsymbol{\beta}}, \hat{\phi}, \hat{\boldsymbol{\tau}}|\tilde{\boldsymbol{b}}) + (\boldsymbol{b} - \tilde{\boldsymbol{b}})^\intercal \nabla\ell_{PQL}(\tilde{\boldsymbol{\beta}}, \hat{\phi}, \hat{\boldsymbol{\tau}}|\tilde{\boldsymbol{b}}) + \frac{1}{2}(\boldsymbol{b} - \tilde{\boldsymbol{b}})^\intercal \nabla^2\ell_{PQL}(\tilde{\boldsymbol{\beta}}, \hat{\phi}, \hat{\boldsymbol{\tau}}|\tilde{\boldsymbol{b}})(\boldsymbol{b} - \tilde{\boldsymbol{b}}).$$

This leads to the Newton's updates

$$\begin{aligned}\hat{\boldsymbol{b}} &= \tilde{\boldsymbol{b}} + \left[-\nabla^2_{\tilde{\boldsymbol{b}}}\ell_{PQL}(\tilde{\boldsymbol{\beta}}, \hat{\phi}, \hat{\boldsymbol{\tau}}|\tilde{\boldsymbol{b}})\right]^{-1} \nabla_{\tilde{\boldsymbol{b}}}\ell_{PQL}(\tilde{\boldsymbol{\beta}}, \hat{\phi}, \hat{\boldsymbol{\tau}}|\tilde{\boldsymbol{b}}) \\ &= \tilde{\boldsymbol{b}} + \left(\boldsymbol{W} + \left(\sum_{s=1}^{S} \hat{\tau}_s \boldsymbol{V}_s\right)^{-1}\right)^{-1} \left(\boldsymbol{W}\Delta(\boldsymbol{y} - \boldsymbol{\mu}) - \left(\sum_{s=1}^{S} \hat{\tau}_s \boldsymbol{V}_s\right)^{-1} \tilde{\boldsymbol{b}}\right) \\ &= \left(\sum_{s=1}^{S} \hat{\tau}_s \boldsymbol{V}_s\right) \left(\boldsymbol{W}^{-1} + \sum_{s=1}^{S} \hat{\tau}_s \boldsymbol{V}_s\right)^{-1} \left(\Delta(\boldsymbol{y} - \boldsymbol{\mu}) + \tilde{\boldsymbol{b}}\right). \end{aligned} \tag{11}$$

Defining the working vector $\tilde{\boldsymbol{Y}}$ with elements $\tilde{Y}_i = \eta_i + g'(\mu_i)(y_i - \mu_i)$, the solution of (11) is equal to

$$\hat{\boldsymbol{b}} = \left(\sum_{s=1}^{S} \hat{\tau}_s \boldsymbol{V}_s\right) \boldsymbol{\Sigma}^{-1} \left(\tilde{\boldsymbol{Y}} - \tilde{\boldsymbol{X}}\tilde{\boldsymbol{\beta}}\right), \tag{12}$$

where $\tilde{\boldsymbol{X}} = [\boldsymbol{X} \quad \boldsymbol{G}]$ and $\boldsymbol{\Sigma} = \boldsymbol{W}^{-1} + \sum_{s=1}^{S} \hat{\tau}_s \boldsymbol{V}_s$. Because the weights $\boldsymbol{W}$ are being updated repeatedly, the solution (12) requires inverting a different variance-covariance matrix $\boldsymbol{\Sigma}$ at each iteration, with complexity $O(n^3)$. In modern large-scale data sets, the sample size n can be very large, thus we want to avoid costly matrix inversions. For binary traits, we have that

$$\nabla^2_{\tilde{\boldsymbol{b}}}\ell_{PQL}(\tilde{\boldsymbol{\beta}}, \hat{\phi}, \hat{\boldsymbol{\tau}}|\tilde{\boldsymbol{b}}) \succeq -0.25\boldsymbol{I}_n - \left(\sum_{s=1}^{S} \hat{\tau}_s \boldsymbol{V}_s\right)^{-1}.$$

Therefore, we can replace the hessian by its lower-bound in the quadratic approximation $f(\boldsymbol{b})$ (Böhning and Lindsay, 1988). This leads to a minorization-maximization (MM) algorithm (Hunter and Lange, 2004) with updates

$$\begin{aligned}\hat{\boldsymbol{b}} &= \tilde{\boldsymbol{b}} + \left(0.25\boldsymbol{I}_n + \left(\sum_{s=1}^{S} \hat{\tau}_s \boldsymbol{V}_s\right)^{-1}\right)^{-1} \left(\boldsymbol{W}\Delta(\boldsymbol{y} - \boldsymbol{\mu}) - \left(\sum_{s=1}^{S} \hat{\tau}_s \boldsymbol{V}_s\right)^{-1} \tilde{\boldsymbol{b}}\right) \\ &= \left(\sum_{s=1}^{S} \hat{\tau}_s \boldsymbol{V}_s\right) \left(4\boldsymbol{I}_n + \sum_{s=1}^{S} \hat{\tau}_s \boldsymbol{V}_s\right)^{-1} \left(4(\boldsymbol{y} - \boldsymbol{\mu}) + \tilde{\boldsymbol{b}}\right). \end{aligned} \tag{13}$$

Redefining the working vector $\tilde{\boldsymbol{Y}}$ with elements $\tilde{Y}_i = \eta_i + 4(y_i - \mu_i)$, the solution of (13) is equal to

$$\hat{\boldsymbol{b}} = \left(\sum_{s=1}^{S} \hat{\tau}_s \boldsymbol{V}_s\right) \tilde{\boldsymbol{\Sigma}}^{-1} \left(\tilde{\boldsymbol{Y}} - \tilde{\boldsymbol{X}}\tilde{\boldsymbol{\beta}}\right), \tag{14}$$

where $\tilde{\boldsymbol{\Sigma}} = 4\boldsymbol{I}_n + \sum_{s=1}^{S} \hat{\tau}_s \boldsymbol{V}_s$.

Let $\sum_{s=1}^{S} \hat{\tau}_s \boldsymbol{V}_s = \boldsymbol{U}\boldsymbol{D}\boldsymbol{U}^\intercal$ be the associated eigen-spectral decomposition of the variance-covariance matrix of $\boldsymbol{b}$, where $\boldsymbol{U}_{n\times n}$ is an orthonormal matrix of eigenvectors and $\boldsymbol{D}_{n\times n}$ is a diagonal matrix of

eigenvalues, such that (14) can be rewritten as

$$\hat{\boldsymbol{b}} = \boldsymbol{U}\left(4\boldsymbol{D}^{-1} + \boldsymbol{I}_n\right)^{-1}\boldsymbol{U}^\intercal\left(\tilde{\boldsymbol{Y}} - \tilde{\boldsymbol{X}}\tilde{\boldsymbol{\beta}}\right). \tag{15}$$

By rotating the random effect $\boldsymbol{\delta} = \boldsymbol{U}^\intercal \boldsymbol{b}$, we have that (15) is equivalent to solving the following generalized ridge regression problem

$$\hat{\boldsymbol{\delta}} = \underset{\boldsymbol{\delta}}{\operatorname{argmin}}\ \frac{1}{4}\left(\tilde{\boldsymbol{Y}} - \tilde{\boldsymbol{X}}\tilde{\boldsymbol{\beta}} - \boldsymbol{U}\boldsymbol{\delta}\right)^\intercal\left(\tilde{\boldsymbol{Y}} - \tilde{\boldsymbol{X}}\tilde{\boldsymbol{\beta}} - \boldsymbol{U}\boldsymbol{\delta}\right) + \boldsymbol{\delta}^\intercal\boldsymbol{D}^{-1}\boldsymbol{\delta}.$$

Consider now a coordinate descent step for $\boldsymbol{\beta}$. That is, suppose we have updates $\tilde{\boldsymbol{b}}$ and $\tilde{\boldsymbol{\beta}}_l$ for $l \neq j$, and we wish to partially optimize with respect to β_j. We would like to compute the gradient at $\beta_j = \tilde{\beta}_j$, which only exists if $\tilde{\beta}_j \neq 0$. If $\tilde{\beta}_j > 0$, then

$$\frac{\partial Q_\lambda(\boldsymbol{\beta}, \boldsymbol{b})}{\partial \beta_j}\Big|_{(\boldsymbol{b},\boldsymbol{\beta})=(\tilde{\boldsymbol{b}},\tilde{\boldsymbol{\beta}})} = -\sum_{i=1}^{n}\frac{a_i(y_i - \mu_i)}{\hat{\phi}\nu(\mu_i)}\frac{1}{g'(\mu_i)}\tilde{X}_{ij} + \lambda v_j = -\tilde{\boldsymbol{X}}_j^\intercal \boldsymbol{W}\Delta(\boldsymbol{y} - \boldsymbol{\mu}) + \lambda v_j, \tag{16}$$

where $\tilde{\boldsymbol{X}}_j$ is a $n \times 1$ column vector for predictor j. Recall that for binary traits, we defined the working vector $\tilde{\boldsymbol{Y}}$ such that $\boldsymbol{y} - \boldsymbol{\mu} = \frac{1}{4}(\tilde{\boldsymbol{Y}} - \tilde{\boldsymbol{X}}\boldsymbol{\beta} - \boldsymbol{b})$. Moreover, for binary traits with logistic link function, we have $\phi = 1$ and $\boldsymbol{W} = \boldsymbol{\Delta}^{-1}$. Thus, plugging $\tilde{\boldsymbol{b}} = \hat{\boldsymbol{b}}$ from (15) and solving (16) leads to

$$\begin{aligned}
&-\frac{1}{4}\tilde{\boldsymbol{X}}_j^\intercal\left(\tilde{\boldsymbol{Y}} - \tilde{\boldsymbol{X}}\tilde{\boldsymbol{\beta}} - \tilde{\boldsymbol{b}}\right) + \lambda v_j = 0\\
\iff &-\frac{1}{4}\tilde{\boldsymbol{X}}_j^\intercal\boldsymbol{U}\left(\boldsymbol{I}_n - \left(4\boldsymbol{D}^{-1} + \boldsymbol{I}_n\right)^{-1}\right)\boldsymbol{U}^\intercal\left(\tilde{\boldsymbol{Y}} - \tilde{\boldsymbol{X}}\tilde{\boldsymbol{\beta}}\right) + \lambda v_j = 0\\
\iff &-\tilde{\boldsymbol{X}}_j^\intercal\boldsymbol{U}\left(4\boldsymbol{I}_n + \boldsymbol{D}\right)^{-1}\boldsymbol{U}^\intercal\left(\tilde{\boldsymbol{Y}} - \tilde{\boldsymbol{X}}\tilde{\boldsymbol{\beta}}\right) + \lambda v_j = 0.
\end{aligned}$$

Finally, isolating β_j yields

$$\begin{aligned}
\hat{\beta}_j &= \frac{\tilde{\boldsymbol{X}}_j{}^\intercal\boldsymbol{U}\left(4\boldsymbol{I}_n + \boldsymbol{D}\right)^{-1}\boldsymbol{U}^\intercal\left(\tilde{\boldsymbol{Y}} - \sum_{l\neq j}\tilde{\boldsymbol{X}}_l\tilde{\beta}_l\right) - \lambda v_j}{\tilde{\boldsymbol{X}}_j^\intercal\boldsymbol{U}\left(4\boldsymbol{I}_n + \boldsymbol{D}\right)^{-1}\boldsymbol{U}^\intercal\tilde{\boldsymbol{X}}_j}\\
&= \frac{\sum_{i=1}^{n}\frac{1}{4+\Lambda_i}\tilde{X}_{ij}^{*}\left(\tilde{Y}_i^{*} - \sum_{l\neq j}\tilde{X}_{il}^{*}\tilde{\boldsymbol{\beta}}_l\right) - \lambda v_j}{\sum_{i=1}^{n}\frac{1}{4+\Lambda_i}\tilde{X}_{ij}^{*2}},
\end{aligned} \tag{17}$$

where Λ_i are the eigenvalues of $\sum_{s=1}^{S}\hat{\tau}_s\boldsymbol{V}_s$, $\tilde{\boldsymbol{Y}}^* = \boldsymbol{U}^\intercal\tilde{\boldsymbol{Y}}$ and $\tilde{\boldsymbol{X}}^* = \boldsymbol{U}^\intercal\tilde{\boldsymbol{X}}$. By proceeding in a similar way for $\tilde{\beta}_j < 0$, one can show (Friedman et al., 2007) that the coordinate-wise update for β_j has the form

$$\hat{\beta}_j = \frac{S\left(\sum_{i=1}^{n}\frac{1}{4+\Lambda_i}\tilde{X}_{ij}^{*}\left(\tilde{Y}_i^{*} - \sum_{l\neq j}\tilde{X}_{il}^{*}\tilde{\boldsymbol{\beta}}_l\right), \lambda v_j\right)}{\sum_{i=1}^{n}\frac{1}{4+\Lambda_i}\tilde{X}_{ij}^{*2}}, \tag{18}$$

where $S(z,\gamma)$ is the soft-thresholding operator:

$$\text{sign}(z)(|z|-\gamma)_+ = \begin{cases} z-\gamma & \text{if } z>0 \text{ and } \gamma<|z| \\ z+\gamma & \text{if } z<0 \text{ and } \gamma<|z| \\ 0 & \text{if } \gamma \geq |z|. \end{cases}$$

Finally, the updates for $\boldsymbol{\eta}$ are given by

$$\begin{aligned} \hat{\boldsymbol{\eta}} &= \tilde{\boldsymbol{Y}} - \left(\tilde{\boldsymbol{Y}} - \boldsymbol{X}\hat{\boldsymbol{\beta}} - \hat{\boldsymbol{b}}\right) \\ &= \tilde{\boldsymbol{Y}} - \boldsymbol{U}\left\{\boldsymbol{I}_n - (4\boldsymbol{D}^{-1}+\boldsymbol{I}_n)^{-1}\right\}\left(\tilde{\boldsymbol{Y}}^* - \tilde{\boldsymbol{X}}^*\boldsymbol{\beta}\right) \\ &= \tilde{\boldsymbol{Y}} - \boldsymbol{U}\left(\frac{1}{4}\boldsymbol{D}+\boldsymbol{I}_n\right)^{-1}\left(\tilde{\boldsymbol{Y}}^* - \tilde{\boldsymbol{X}}^*\hat{\boldsymbol{\beta}}\right). \end{aligned} \tag{19}$$

We performed additional simulations in Appendix D and show that coefficients estimates for $\boldsymbol{\beta}$ obtained by replacing the hessian by a lower bound are similar to those obtained by repeatedly inverting the full hessian matrix at each iteration.

The cyclic coordinate descent algorithm to obtain regularized PQL estimates for $\boldsymbol{\beta} = (\boldsymbol{\alpha}^\intercal, \boldsymbol{\gamma}^\intercal)^\intercal$ and $\boldsymbol{b}$ is as follows:

Algorithm 2: Cyclic coordinate descent for regularized PQL estimation

---

1. *Initialization*

   Set $\hat{\boldsymbol{\beta}}^{(0)} = (\hat{\boldsymbol{\alpha}}^\intercal, \boldsymbol{0}^\intercal)$ and $\hat{\boldsymbol{b}}^{(0)} = \hat{\boldsymbol{b}}$, where $\hat{\boldsymbol{\alpha}}, \hat{\boldsymbol{b}}$ are the estimates from the AI-REML algorithm;

   Calculate $\hat{\boldsymbol{\eta}}^{(0)} = \tilde{\boldsymbol{X}}\hat{\boldsymbol{\beta}}^{(0)} + \hat{\boldsymbol{b}}^0$, $\tilde{\boldsymbol{Y}}^{*(0)} = \boldsymbol{U}^\intercal\tilde{\boldsymbol{Y}}^{(0)}$ and $\tilde{\boldsymbol{X}}^* = \boldsymbol{U}^\intercal\tilde{\boldsymbol{X}}$;

2. *Iteration*

   for $\lambda = \lambda_{max}$ to λ_{min} **do**

       for $t = 1, 2, ...,$ until outer-loop convergence **do**

           for $j = 1, ..., m+p$ **do**

               Calculate

$$\hat{\boldsymbol{\beta}}_j^{(t)} = \frac{S\left(\sum_{i=1}^n \frac{1}{4+\Lambda_i}\tilde{X}_{ij}^*\left(\tilde{Y}_i^{*(t-1)} - \sum_{l\neq j}\tilde{X}_{il}^*\hat{\boldsymbol{\beta}}_l^{(t-1)}\right), \lambda v_j\right)}{\sum_{i=1}^n \frac{1}{4+\Lambda_i}\tilde{X}_{ij}^{*2}},$$

               until inner-loop convergence;

           Calculate $\hat{\boldsymbol{\eta}}^{(t)} = \tilde{\boldsymbol{Y}}^{(t-1)} - \boldsymbol{U}\left(\frac{1}{4}\boldsymbol{D}+\boldsymbol{I}_n\right)^{-1}\left(\tilde{\boldsymbol{Y}}^{*(t-1)} - \tilde{\boldsymbol{X}}^*\hat{\boldsymbol{\beta}}^{(t)}\right)$;

           Update $\tilde{\boldsymbol{Y}}^{(t)}$ and $\tilde{\boldsymbol{Y}}^{*(t)}$ using $\hat{\boldsymbol{\eta}}^{(t)}$;

       Set $\hat{\boldsymbol{\beta}}^{(0)} = \hat{\boldsymbol{\beta}}^{(t)}$, $\tilde{\boldsymbol{Y}}^{(0)} = \tilde{\boldsymbol{Y}}^{(t)}$ and $\tilde{\boldsymbol{Y}}^{*(0)} = \tilde{\boldsymbol{Y}}^{*(t)}$ as warm starts for next λ;

For inner-loop convergence, we use the same criteria as Friedman et al. (2007), that is after a complete cycle of coordinate descent we look at

$$\max_j \Delta_j = \max_j \sum_{i=1}^{n} \frac{1}{4+\Lambda_i} \tilde{X}_{ij}^{*2} (\hat{\beta}_j^{(t-1)} - \hat{\beta}_j^{(t)})^2,$$

which measures the maximum weighted sum of squares of changes in fitted values for all coefficients. If $\max_j \Delta_j$ is smaller than tolerance, we stop the coordinate descent loop. For outer-loop convergence, we calculate the fractional change in the loss function $-l_{PQL}(\boldsymbol{\alpha}, \boldsymbol{\gamma}, \hat{\phi}, \hat{\boldsymbol{\tau}})$ and declare convergence if its value is smaller than tolerance.

Appendix C. Model selection

Approaches to selecting the optimal tuning parameter in regularized models are of primary interest since in real data analysis, the underlying true model is unknown. A popular strategy is to select the value that minimizes out-of-sample prediction error, e.g., cross-validation (CV), which is asymptotically equivalent to the Akaike information criterion (AIC) (Akaike, 1998; Yang, 2005). While being conceptually attractive, CV becomes computationally expensive for very high-dimensional data. Moreover, in studies where the proportion of related subjects is important, either by known or cryptic relatedness, the CV prediction error is no longer an unbiased estimator of the generalization error (Rabinowicz and Rosset, 2020). Through simulation studies and real data analysis, Wang et al. (2020) found that LD and minor allele frequencies (MAF) differences between ancestries could explain between 70 and 80% of the loss of relative accuracy of European-based prediction models in African ancestry for traits like body mass index and type 2 diabetes. Thus, there is no clear approach to how multiple admixed and/or similar populations should be split when using CV to minimize out-of-sample prediction error.

Alternatively, we can select the optimal value of the tuning parameter by optimizing the generalized information criterion (GIC) with an appropriate model complexity penalty a_n, defined as

$$\text{GIC}_\lambda = -2\ell_{PQL} + a_n \cdot \hat{df}_\lambda, \tag{20}$$

where ℓ_{PQL} is defined in (3), and $\hat{df}_\lambda = |\{1 \leq k \leq p : \hat{\beta}_k \neq 0\}| + \dim(\hat{\boldsymbol{\tau}})$ is the number of nonzero fixed-effects coefficients (Zou et al., 2007) plus the number of variance components. The choice of a_n becomes crucial for effectively identifying the true model in high-dimensional data. Fan and Tang (2012) have proposed using a high-dimensional Bayesian information criterion (HDBIC) with $a_n = \log(\log n)\log p$. However, in our simulations and analysis of real data, our findings were that using $a_n = 2$ (AIC) was an appropriate model complexity penalty, and that using $a_n = \log(n)$ (BIC, Schwarz (1978)) resulted in sparse models with almost no predictors. Hence, we did not investigate in this work the use of more

severe model complexity penalties such as the HDBIC, but in our software implementation we allow to choose between AIC, BIC and HDBIC for selecting the best model.

Appendix D. Comparison of coefficient estimates using a lower-bound algorithm

We performed additional simulations to evaluate the impact on the coefficient estimates of taking a lower-bound of the variance-covariance matrix. More specifically, we simulated random genotypes from the BN-PSD admixture model for 10 intermediate populations of the 1D linear admixture model, with a total of $n = 2500$ samples, $p = 5000$ candidate SNPs where we randomly selected $c = 1\%$ to be causal. We fitted the full lasso path for 100 values of the regularization parameter λ. We report in Figure 1 the median mean squared-error (MSE) for 20 replications, defined as $\text{MSE}(\hat{\boldsymbol{\beta}}) = \|\hat{\boldsymbol{\beta}} - \boldsymbol{\beta}\|^2/p$, when we repeatedly inverted the full variance-covariance matrix (`pglmm_hessian`), and when we replaced the hessian by a lower-bound (`pglmm`). We also included in the comparison the coefficient estimates for a logistic lasso with 10 PCs (`glmnetPC`). We can see that all 3 methods lead to similar estimates on the full lasso path as measured by the median $\text{MSE}(\hat{\boldsymbol{\beta}})$. For models with large number of active predictors, the estimates based on the lower-bound method are closer to the true estimates compared to the other methods. We note that for binary responses, the existing lower-bound on the variance-covariance matrix not only provides an increasing computational advantage over Newton–Raphson, but also guarantees linear convergence to the optimal solution (Böhning and Lindsay, 1988). Thus, using a lower-bound on the hessian of the loss function for logistic regression is commonly done in majorization-minimization (MM) algorithms (Hu et al., 2019; Hunter and Lange, 2004).

Figure 1: Median MSE($\hat{\boldsymbol{\beta}}$) for 20 replications of the simulated genotype with 1d linear admixture and $K = 10$ subpopulations. Compared methods are `pglmm` with a lower-bound algorithm, `pglmm_hessian` where we repeatedly invert the full variance-covariance matrix and logistic lasso with 10 PCs (`glmnetPC`).

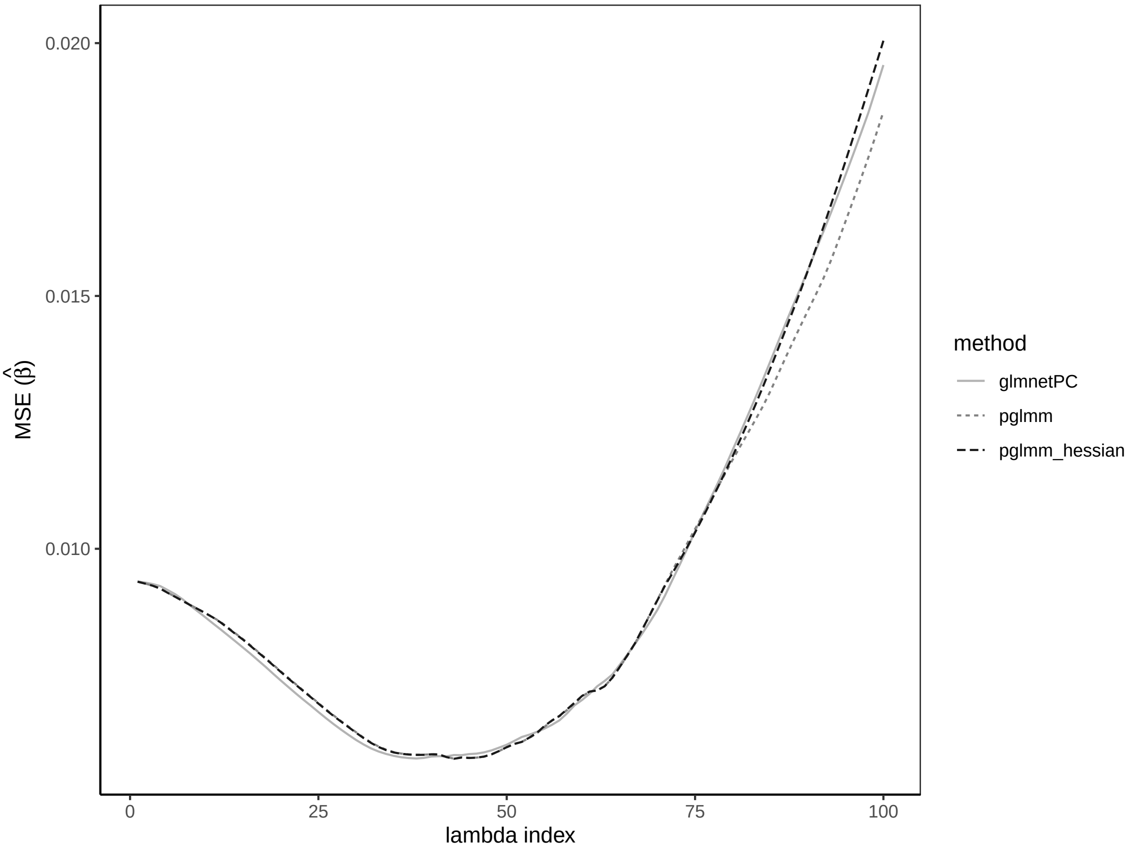

Appendix E. Confounding from population structure

Figure 2: Correlation heatmap between the first 20 PCs and $K = 20$ indicator functions identifying the independent subpopulations from the simulated genotype. We used the absolute value or the Pearson's correlation coefficient for the color scaling, and displayed the value whenever $|r^2| > 0.2$.

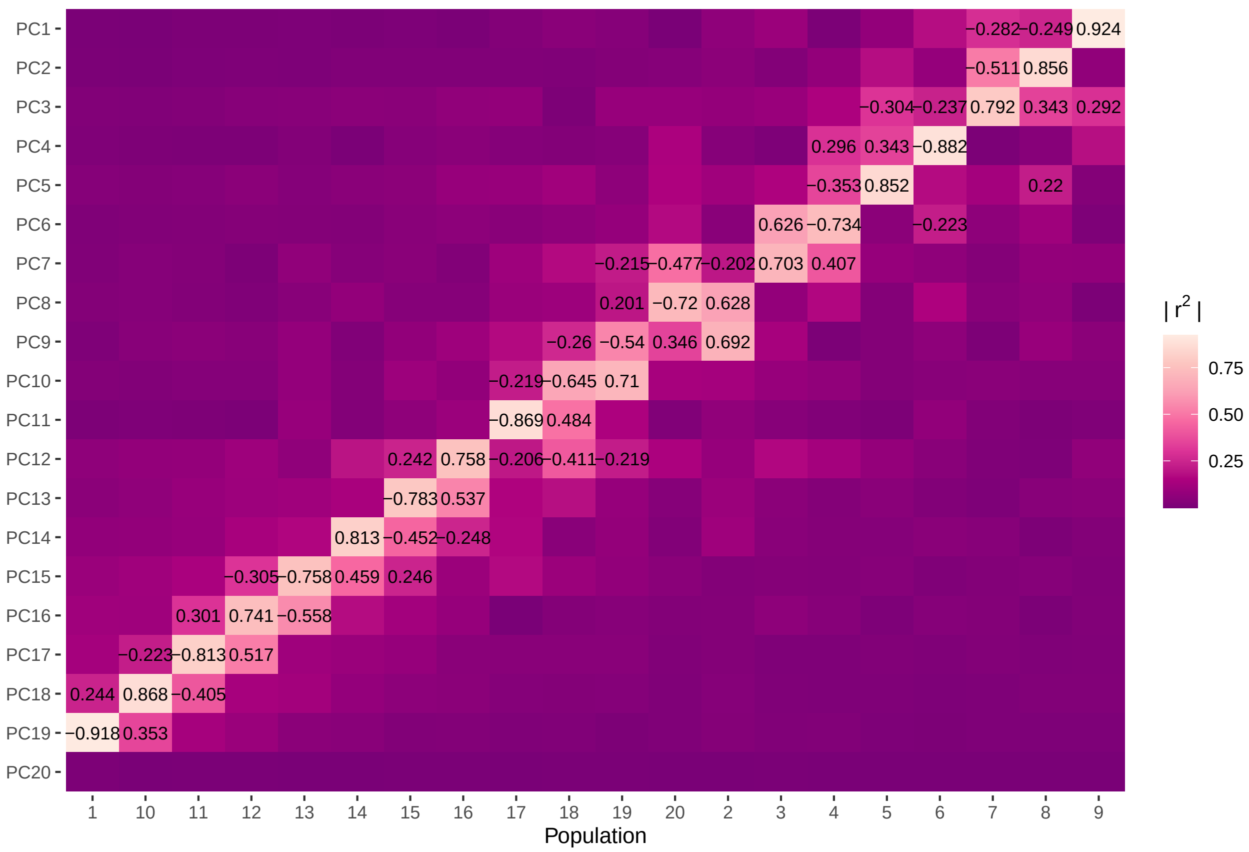

Figure 3: Correlation heatmap between the first 20 PCs and $K = 20$ indicator functions identifying subpopulations from the simulated genotype with 1d linear admixture. We used the absolute value or the Pearson's correlation coefficient for the color scaling, and displayed the value whenever $|r^2| > 0.2$.

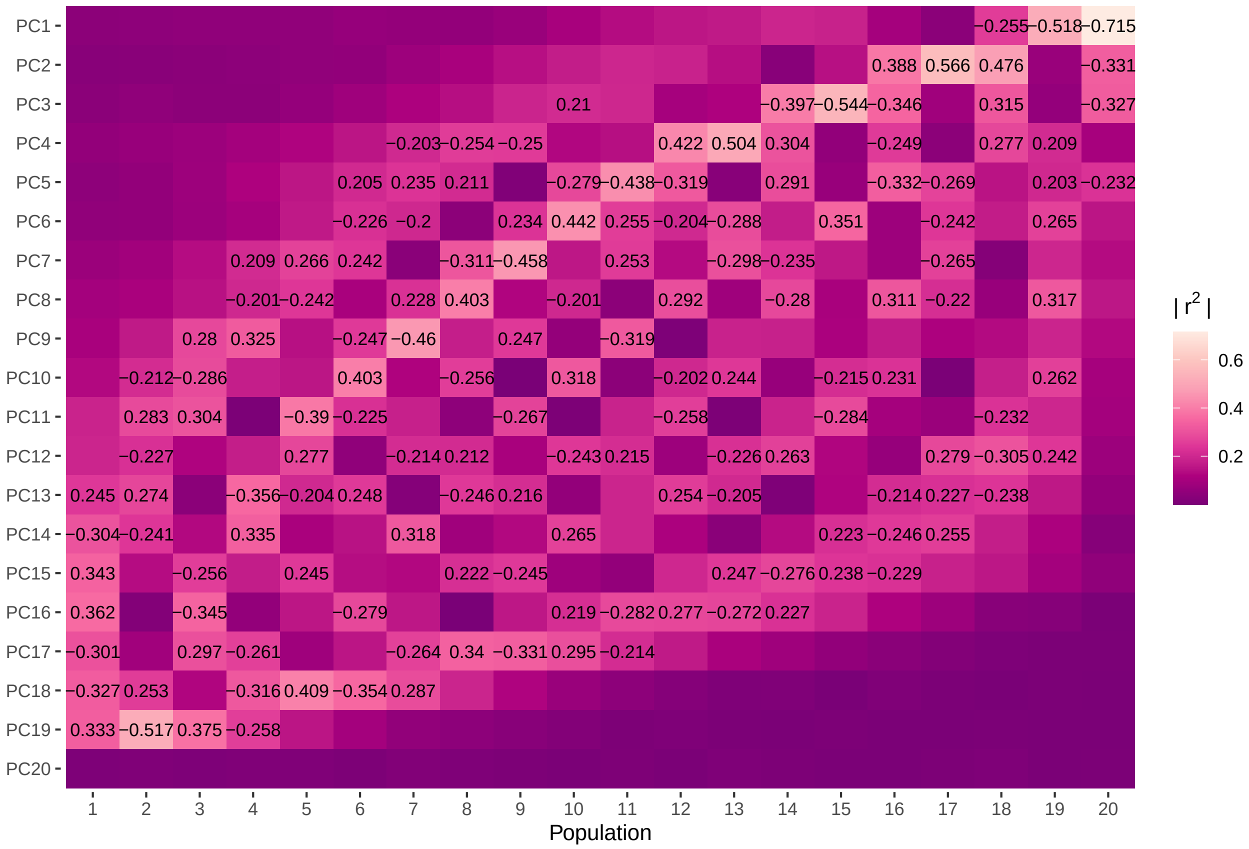

References

Akaike, H. (1998). Information Theory and an Extension of the Maximum Likelihood Principle, pages 199–213. Springer New York, New York, NY. 9

Böhning, D. and Lindsay, B. G. (1988). Monotonicity of quadratic-approximation algorithms. Annals of the Institute of Statistical Mathematics, 40(4):641–663. 6, 10

Chen, H., Wang, C., Conomos, M. P., Stilp, A. M., Li, Z., Sofer, T., Szpiro, A. A., Chen, W., Brehm, J. M., Celedón, J. C., Redline, S., Papanicolaou, G. J., Thornton, T. A., Laurie, C. C., Rice, K., and Lin, X. (2016). Control for population structure and relatedness for binary traits in genetic association studies via logistic mixed models. The American Journal of Human Genetics, 98(4):653–666. 2

Fan, Y. and Tang, C. Y. (2012). Tuning parameter selection in high dimensional penalized likelihood. Journal of the Royal Statistical Society: Series B (Statistical Methodology), 75(3):531–552. 9

Friedman, J., Hastie, T., Höfling, H., and Tibshirani, R. (2007). Pathwise coordinate optimization. The Annals of Applied Statistics, 1(2). 7, 9

Hu, L., Lu, W., Zhou, J., and Zhou, H. (2019). MM ALGORITHMS FOR VARIANCE COMPONENT ESTIMATION AND SELECTION IN LOGISTIC LINEAR MIXED MODEL. Statistica Sinica. 10

Hunter, D. R. and Lange, K. (2004). A tutorial on MM algorithms. The American Statistician, 58(1):30–37. 6, 10

Rabinowicz, A. and Rosset, S. (2020). Cross-validation for correlated data. Journal of the American Statistical Association, pages 1–14. 9

Schwarz, G. (1978). Estimating the dimension of a model. The Annals of Statistics, 6(2). 9

Wang, Y., Guo, J., Ni, G., Yang, J., Visscher, P. M., and Yengo, L. (2020). Theoretical and empirical quantification of the accuracy of polygenic scores in ancestry divergent populations. Nature Communications, 11(1). 9

Yang, Y. (2005). Can the strengths of aic and bic be shared? a conflict between model indentification and regression estimation. Biometrika, 92(4):937–950. 9

Zou, H., Hastie, T., and Tibshirani, R. (2007). On the "degrees of freedom" of the lasso. The Annals of Statistics, 35(5). 9
